# Pulmonary hypertension: etiology and anti-inflammatory treatment pathways of natural products

**DOI:** 10.3389/fphar.2025.1743782

**Published:** 2026-01-26

**Authors:** Liyang Li, Xing Huang, Maojun Cheng, Chengxun He, Changmao Dai, Fang Ding, Jia Xu, Feier Chen, Kaichen Zhang, Xueping Li

**Affiliations:** 1 Hosptial of Chengdu University of Traditional Chinese Medicine, Chengdu, Sichuan, China; 2 School of Clinical Medicine, Chengdu University of Traditional Chinese Medicine, Chengdu, Sichuan, China; 3 Leshan Traditional Chinese Medicine Hospital, Leshan, Sichuan, China

**Keywords:** etiology, inflammation, mechanisms, natural products, pulmonary hypertension

## Abstract

Pulmonary hypertension (PH) is a severe and progressive cardiopulmonary disorder that affects multiple organ systems. Clinically, it is manifested by progressive dyspnea, which progressively worsens with exertion and ultimately results in right heart failure and death at a late stage. Its pathological features are primarily characterized by abnormally elevated pulmonary artery pressure, pulmonary vascular remodeling, and thrombus formation. The inflammatory response is widely recognized as a key initiating factor and critical component in the progression of PH. Furthermore, therapeutic interventions are hampered by the irreversibility of vascular remodeling, high treatment expense, and hepatotoxicity induced by pharmacologic agents. Therefore, it is of great importance and potential to develop novel therapies with multi-targeted, safe, efficacious, and low-cost characteristics. Emerging evidence has demonstrated that natural products (NPs) possess remarkable potential in modulating pulmonary vascular remodeling (PVR) and right ventricular function, and their effects may be associated with inflammation. In this review, we comprehensively review recent advances in the use of NPs to ameliorate PH by modulating inflammation-associated signaling pathways, highlighting the crucial role of inflammation in PH pathogenesis. We attempt to provide a theoretical basis and research strategy for using inflammatory approaches with natural products in PH, which would be helpful for the development of novel therapies.

## 1 Introduction

Pulmonary hypertension (PH) is a clinically serious and progressive disorder, characterized pathologically by pulmonary arteriolar vasoconstriction, vascular remodeling, and thrombus formation (Humbert et al., 2023). Patients with pulmonary hypertension frequently present clinically with progressive dyspnoea and exercise intolerance, ultimately progressing to right-sided heart failure and potentially fatal outcomes (Johnson et al., 2023). According to the classification, PH can be subdivided into five types, including pulmonary arterial hypertension (PAH); pulmonary hypertension associated with left-sided heart disease; PH caused by lung disease or hypoxia; PH associated with pulmonary arterial obstructions—usually caused by thromboembolic disease; and PH with unclear and/or multifactorial mechanisms. In 2022, the guidelines published by the European Society of Cardiology (ESC) and the European Respiratory Society (ERS) defined PH as a progressive disorder with the mean pulmonary arterial pressure above 20 mmHg at rest (Humbert et al., 2023). Epidemiological reports suggested that PH affects about 1%–3% of the general population, and the prevalence was nearly 10% in adults over 65 years of age, indicating that PH has become a great public health problem (Hoeper et al., 2016).

During recent decades, pharmacological therapies that target the pathophysiology of PH have emerged one after the other, for instance, calcium channel blockers, prostacyclin analogs, endothelin receptor antagonists (ERAs), phosphodiesterase type 5 (PDE5) inhibitors, and soluble guanylate cyclase (sGC) stimulators (Hoeper et al., 2017; Lajoie et al., 2016; Zolty, 2020). Although these medicines could ameliorate the symptoms of selected patients, many challenges remain, such as the irreversible pulmonary vascular remodeling, drug-induced hepatotoxicity, and high cost of these medicines for long-term use, which bring a heavy economic burden to patients and healthcare systems (Yorifuji et al., 2020; Watzker et al., 2025). Therefore, it is of great urgency to find new therapeutic strategies with multi-targeted, safe, effective, and economically friendly properties. In this regard, natural products (NPs), which are derived from plants, microorganisms, animals, and marine organisms, have attracted increasing attention. With multi-target activity and relatively low toxicity, NPs might provide novel therapies with therapeutic advantages for the complex pathophysiology of PH (Wang X. et al., 2024; Zhang J. J. et al., 2024).

**FIGURE 1:**
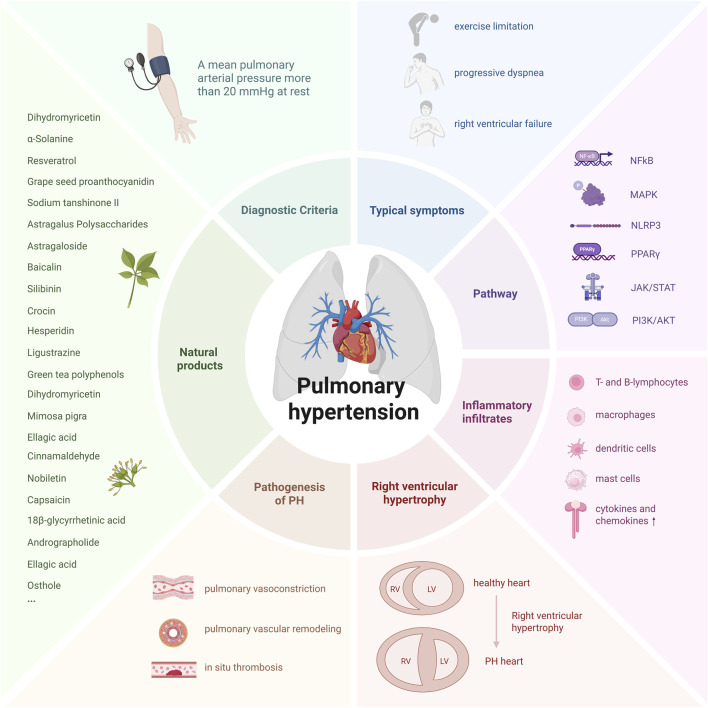
The primary biological phenomena and pathological characteristics of PH. (Created with BioRender-YI295TYAWQ).

Among the multiple mechanisms leading to PH, inflammation is currently considered a critical initial event and a pivotal regulator in the progression of disease (Klouda and Yuan, 2021). A large body of evidence supports that in pulmonary hypertension, pro-inflammatory cytokines and inflammatory cell infiltration in the lungs trigger endothelial dysfunction, PASMC proliferation, and ECM remodeling, ultimately leading to irreversible pulmonary vascular remodeling (Tsuboya et al., 2025). Growing evidence has demonstrated that NPs could exhibit remarkable effects in ameliorating pulmonary vascular remodeling (PVR) and protecting right ventricular function, which are closely related to the modulation of inflammatory responses (Shi et al., 2018a; Zeng et al., 2023). Therefore, NPs have a great potential as a therapeutic agent for PH. In this review, we aim to systematically elucidate the central role of inflammation in PH pathogenesis and summarize the recent progress in NP-based therapeutic strategies targeting inflammatory signaling pathways. We hope that this review could provide a theoretical basis and research ideas for the development of anti-inflammatory signaling pathway targeting PH using NP, as well as discovering novel therapeutic agents (Figure 1).

## 2 Methodology

To gain a deeper insight into the complicated cross-talk among NP, inflammation, and PH, we performed a thorough literature mining from different medical databases. In order to ensure the comprehensiveness and reliability of literature mining, we selected 4 major databases, PubMed, Web of Science, ScienceDirect, and Google Scholar, for retrieving literature and collecting references. The searching keywords were “pulmonary arterial hypertension,” “pulmonary hypertension,” “mechanism,” “natural products,” “traditional Chinese medicine,” “medicinal plants,” “inflammation,” “inflammatory response”, “chronic inflammation”, “acute inflammation”, etc, and different combinations of the above searching keywords. Based on the above-mentioned literature screening, we further analyzed the selected high-relevance literature in depth. Inclusion criteria: (1) Original research articles, (2) The research subjects are diseases associated with pulmonary arterial hypertension, or key pulmonary vascular cells whose induction conditions correlate with the pathology of pulmonary hypertension, (3) The study will include literature published between January 2012 and the date of retrieval. Exclusion criteria: (1) Reviews, editorials, conference abstracts/abstracts only, programme papers, case reports/small-sample studies without controls; (2) The absence of a necessary control group or key outcome data rendered extraction impossible; (3) Non-English research articles. Through mining and integrating their main data, we summarized the main experimental conclusions and results about the effects of different NPs in models of PH and finally displayed them in tabular form.

## 3 Pathophysiological processes of pulmonary hypertension

**FIGURE 2:**
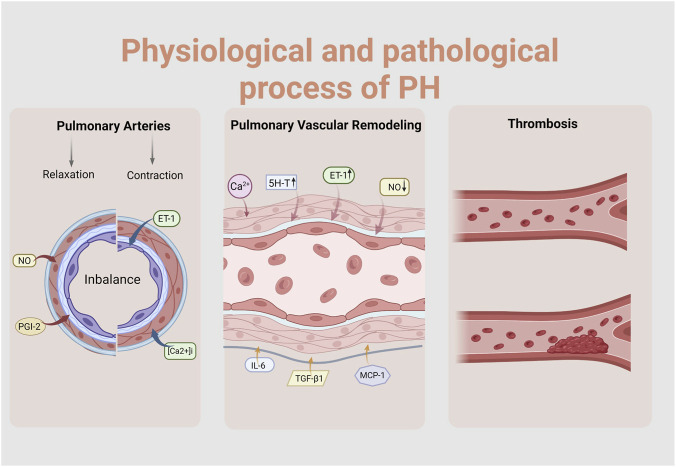
The physiological and pathological process of PH. (Created with BioRender-LS295TWSLP).

Pulmonary hypertension (PH) is a multifactorial disease characterized by structural and functional changes in the pulmonary vasculature, including vasoconstriction, increased vascular resistance, and thrombosis, leading to progressive occlusion of small and medium pulmonary arteries (Poch and Mandel, 2021). This is driven by continuous inflammation, immune dysregulation, abnormal proliferation/apoptosis signaling, EndMT, and genetic factors (e.g., BMPR2 mutation), which promote pulmonary vascular remodeling (Gorelova et al., 2021; Guignabert et al., 2015). This remodeling involves abnormal PASMC proliferation, endothelial dysfunction, apoptosis imbalance, and ECM deposition, resulting in vessel wall thickening and lumen narrowing, further progressing PH (Fu et al., 2025). The pathophysiological process of pulmonary hypertension is illustrated in Figure 2. As pulmonary vascular resistance increases, right ventricular hypertrophy compensates initially, but prolonged overload leads to right ventricular failure, the main cause of death in PH patients (Rosenkranz et al., 2020; Tonelli et al., 2013).

### 3.1 Imbalance between pulmonary vasoconstriction and vasodilation

An imbalance between vasoconstrictive and vasodilatory signaling is a key pathogenic mechanism in PH, mainly involving nitric oxide (NO), prostacyclin, and endothelin-1 (ET-1) pathways as well as changes in the intracellular calcium concentrations (Galiè et al., 2015).

Nitric oxide (NO), synthesized by endothelial NO synthase (eNOS) from L-arginine, diffuses into pulmonary vascular smooth muscle cells (PVSMCs) and activates the soluble guanylate cyclase (sGC)/cGMP/protein kinase G (PKG) pathway, leading to vasodilation and inhibition of smooth muscle proliferation. In PAH, eNOS uncoupling, oxidative stress, and inflammation impair NO bioavailability, promoting excessive PVSMC proliferation and vascular remodeling (Barcena et al., 2022; Murugesan et al., 2022). Similarly, prostacyclin (PGI2), produced from arachidonic acid via cyclooxygenase and prostacyclin synthase, exerts vasodilatory, antiproliferative, and antiplatelet effects; however, in PAH, arachidonic acid is redirected towards the synthesis of vasoconstrictive metabolites such as thromboxane A_2_, rather than being converted into PGI2 (Barnes et al., 2019). The net effect of these mechanisms leads to a reduction of the vasodilatory, antiproliferative, and antiplatelet effects of PGI_2_, which ultimately disrupts pulmonary vascular homeostasis. Conversely, endothelin-1 (ET-1), a potent vasoconstrictor acting through ETA and ETB receptors, becomes overactivated in PAH, stimulating reactive oxygen species production, PVSMC proliferation, and downregulation of the eNOS/sGC pathway. Concurrently, elevated levels of ET-1 exert detrimental effects on vascular remodeling and may exacerbate inflammation (Eid, 2025). Collectively, the downregulation of NO and PGI2 signaling coupled with enhanced ET-1 activity creates a pathological imbalance favoring vasoconstriction, leading to elevated pulmonary vascular resistance and driving disease progression (Christou and Khalil, 2022; Gurzău et al., 2021).

Existing research indicates that various natural products exert potential therapeutic effects against pulmonary arterial hypertension (PAH) by correcting imbalances in vasoconstrictor and vasodilator signaling pathways. These natural metabolite include salidroside, naringenin, total ginsenosides, and kaempferol, and their effects have primarily been evaluated in monocrotaline (MCT)-induced rat models of PAH. Specifically, salidroside (SAL) attenuates PAH progression by modulating arginine metabolism, enhancing endothelial nitric oxide synthase (eNOS) expression and nitric oxide (NO) bioavailability, and activating the soluble guanylate cyclase (sGC)–cyclic guanosine monophosphate (cGMP)–protein kinase G (PKG) pathway (Li et al., 2024). Total ginsenosides (TG) activate the NO/cGMP pathway, inhibit pulmonary vascular remodeling, and ameliorate monocrotaline (MCT)-induced PAH. TG has been shown to enhance eNOS mRNA and protein expression via an Akt-mediated non-genomic activation mechanism, thereby increasing NO and cGMP levels (Lan et al., 2018; Qin et al., 2016). Collectively, SAL and TG alleviate PAH primarily by upstream regulation of NO production. Moreover, SAL reduces endothelin-1 (ET-1) protein levels, thereby relieving transcriptional suppression of the eNOS/sGC signaling pathway, which further enhances NO bioavailability and improves vascular function. Consistent with the ET receptor modulation observed for SAL, blueberry extract (BB) decreases ETA/ETB receptor expression, reduces mean pulmonary arterial pressure (mPAP), and alleviates vasoconstriction and pulmonary vascular remodeling in PAH models (Türck et al., 2020). Moreover, a combined intervention with naringin and L-arginine enhances therapeutic efficacy by not only promoting eNOS expression but also suppressing the overexpression of inducible nitric oxide synthase (iNOS) and the associated inflammatory and proliferative responses, thereby contributing to a more favourable balance between NO sources (eNOS/iNOS) (Ahmed et al., 2014). In contrast to the aforementioned natural products, the flavonoid kaempferol improves right ventricular function and attenuates pulmonary vascular remodeling in PAH rats, primarily by modulating arachidonic acid and amino acid metabolism (Yi et al., 2022).

### 3.2 Pulmonary vascular remodeling

Pulmonary vascular remodeling (PVR) is a major pathologic finding in PH (Jin et al., 2023). Pulmonary vascular remodeling (PVR) is a major pathologic finding in PH, involving pathophysiologic alterations across all three arterial layers—intima, media, and adventitia. This process is characterized by intimal hyperplasia, medial hypertrophy, adventitial fibrosis, and plexiform lesions, leading to progressive luminal narrowing, increased pulmonary vascular resistance, and disrupted balance between vasoconstriction and vasodilation (Yang et al., 2025).

The pulmonary vascular endothelium, consisting of endothelial cells (PAECs) and subendothelium, plays a protective role but becomes dysfunctional in pathological conditions, leading to pulmonary vascular remodeling (PVR) (Chen et al., 2023). Endothelial-to-mesenchymal transition (EndMT), where endothelial cells adopt a smooth muscle-like phenotype, contributes to vascular media thickening and pulmonary hypertension (Yu et al., 2022). Additionally, adventitial fibrosis occurs when fibroblasts become activated, secreting excessive ECM components and attracting inflammatory cells, creating a chronic inflammatory microenvironment that further promotes pulmonary vascular remodeling (Gan et al., 2022; Zhang H. et al., 2024).

In summary, the main characteristics of pulmonary vascular remodeling exhibit various pathological changes, such as pulmonary arterial smooth muscle cell (PASMC) abnormal proliferation, hypertrophy, and migration; endothelial cell (EC) dysfunction and apoptosis resistance; fibroblast activation; ECM over-deposition, and collagen accumulation. These processes are further promoted by environmental factors like chronic inflammation. Finally, it leads to the gradual narrowing or occlusion of the vascular lumen and a gradual and irreversible increase in pulmonary vascular resistance.

### 3.3 Thrombosis

Thrombosis is a frequent pathological finding in PH. This process starts with endothelial dysfunction that induces the activation of the RhoA/Rho kinase pathway, upregulation of tissue factor expression, and the coagulation cascade. This cascade converts prothrombin into thrombin and leads to platelet aggregation (Rose-John et al., 2023).

Under normal physiological conditions, the endothelium of pulmonary arterial vessels exhibits anticoagulant properties. In patients with PH, endothelial anticoagulant function is switched off. NO and prostacyclin, anticoagulant/substance, are downregulated, whereas procoagulant substances such as tissue factor and von Willebrand factor (vWF) are upregulated, leading to a hypercoagulable state and an anti-fibrinolytic microenvironment (Humbert et al., 2014; Manz et al., 2022).

As a chronic inflammatory focus, the thrombi attract and activate pro-inflammatory cells, such as monocytes, macrophages, and platelets (King et al., 2009). The activated platelets release many mediators, including sCD40L, that further induce proliferation, hypertrophy, and endothelial-to-mesenchymal transition (EndMT) of smooth muscle cells. Pathological remodeling reminiscent of PAH is attributable to distal arterioles.

### 3.4 Pulmonary hypertension and inflammation

At different stages of the course of PAH, pulmonary vascular pathology exhibits different degrees of perivascular inflammatory cell infiltration consisting of T and B lymphocytes, macrophages, dendritic cells, and mast cells. This infiltration of immune cells is closely associated with pulmonary vascular remodeling and is undoubtedly an essential part of the pathogenic cascade leading to PH (Zhao et al., 2024). This phenomenon was initially described by Tuder and colleagues in 1994, who found large amounts of inflammatory components around the pulmonary vasculature, including mast cell infiltration in plexiform lesions, increased alveolar macrophages, and peribronchial and perivascular lymphocytic accumulation. Subsequently, experimental studies have found that multiple animal models of PAH exhibited significantly enhanced immune cell infiltration and upregulation of pro-inflammatory mediators (Jin et al., 2021; Wu X. H. et al., 2022). It should be noted that the inflammatory response exists not only in the monocrotaline-induced model, which is the most commonly used experimental model for PAH, but also in other etiological models, indicating that inflammation may be an early pathogenic mechanism and an important factor affecting the development of the disease (Ishibashi et al., 2024; Ricard et al., 2014; Zawia et al., 2021; Xu et al., 2023).

Inflammation is closely related to the pathological process of PAH and is a potential target for treatment. Inflammation is a mediator that unites pulmonary arterial hypertension and autoimmune diseases. So, it is possible to believe that, by inhibiting inflammatory responses, it is possible to find a potential treatment for PH. Natural products (NPs) have attracted extensive attention for their potential uses in health and disease because of their multifunctionality, safety, and cost-effectiveness. Besides, natural products also have many advantages as new substances, such as easy acquisition and almost no side effects. Therefore, natural products are worth exploring as novel applications in health and disease.

## 4 Therapeutic effects of natural products on pulmonary hypertension via inflammatory targets

### 4.1 NF-κB signaling pathway

**FIGURE 3:**
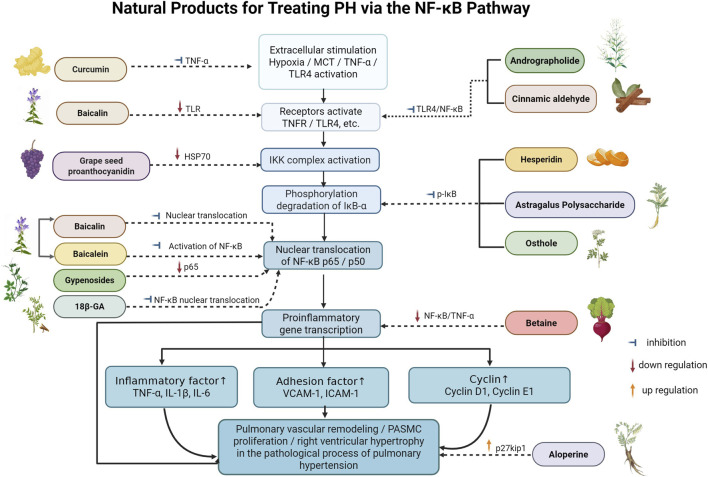
Natural products for treating PH via the NF-κB Pathway (Created with BioRender-DS295TWZI7).

Nuclear factor-κB (NF-κB) is a family of transcription factors involved in the inflammatory response. Additionally, it has been reported that the NF-κB signaling pathway mediates pulmonary vascular remodeling and plays a critical role in the development and progression of PH (Bulgaru et al., 2021; Hosokawa et al., 2013). Many natural products have been reported to relieve an inflammatory state by inhibiting the NF-κB signaling pathway and treating PH, such as hesperidin, baicalin, baicalein, procyanidin from grape seeds, cinnamaldehyde, and andrographolide (Figure 3) (Table 1).

**TABLE 1:** Natural products acting via the NFκB signaling pathway.

Natural products acting via the NFκB signalling pathway								
Natural products	Model used	Species	Model’s induction method	Intervention doses	Administration	Periods	Efficacy	References
Baicalin	*In vivo*	Rat	MCT (60 mg/kg,s.c.)-induced PH rats	Baicalin 100 mg/kg	*i.g*	21 days	Inhibiting PAP, reducing RVH, and attenuating PVR.	Luan et al. (2015)
	*In vivo*	Rat	MCT (50 mg/kg,i.p.)-induced PH rats	Baicalin 20/100/200 mg/kg	*i.p*	30 days	Preventing MCT-induced PAH, pulmonary vascular remodeling, and tissue fibrosis. Reducing the mPAP and RVSP	
	*In vivo*	Rat	MCT (50 mg/kg,i.p.)-induced PH rats	Baicalin 100 mg/kg	*i.g*	6 weeks	Attenuating MCT-induced RVSP, the index of right ventricular hypertrophy, and vessel wall thickness; inhibiting inflammatory and cell proliferation	Yan et al. (2019)
	*In vitro*	PASMCs	TNF-α (5 ng/mL) induced PASMCs	100 μg/mL	*—*	0/24/48/72 h		Xue et al. (2021)
Baicalein	In vivo	Rat	MCT (60 mg/kg,s.c.)-induced PH rats	Baicalein 50/100 mg/kg/day	*i.g*	2 weeks	Alleviating PVR, reducing RVSP, and improving RVH and myocardial cell enlargement	Weiet al. (2018)
	In vivo	Rat	MCT (60 mg/kg,s.c.)-induced PH rats	Baicalein 50/100 mg/kg/day	*i.g*	14 days	Attenuating elevated RVSP, ameliorates RVH and inhibits EndoMT.	R. Shi (D. Zhu (et al. (2018)
Grape seed proanthocyanidin	In vivo	Rat	MCT-induced PH rats	GSP 10 mL/kg	i.p	3 weeks	Decreasing in mPAP, PVR, and RVHI. Inhibiting PASMC proliferation	Chen et al. (2018)
	In vitro	PASMCs	MCT (0.75 g/L)-induced PASMCs	GSP 4 g/L	—	—		
Grape seed procyanidin extract	*In vivo*	Rat	Hypoxic (4 weeks of normoxia or hypoxia exposure) PH rats	GSPE 250 mg/kg/day	*i.g*	4 weeks	GSPE attenuated the elevation of RVSP, RV/LV + S, reduced the pulmonary vascular structure remodeling, and attenuated the proliferation of PASMCs	Jin et al. (2016)
	*In vitro*	PASMCs	Hypoxia-induced PASMCs	GSPE 20/40/80/120 ug/mL	*—*	—		
Cinnamaldehyde	*In vivo*	Rat	SU5416 (20 mg/kg,s.c.)and hypoxic environment-induced PH rats	Cinnamaldehyde 100 mg/kg	*i.g*	3 weeks	*In vivo*: Inhibiting the upregulation of SUV416 and hypoxia-induced RVMP, RVSP, and mPAP in rats, as well as the thickening of the pulmonary artery wall	Zhang et al. (2025)
	*In vitro*	HPAEC	Hypoxia-induced HPAEC	Cinnamaldehyde 10/20/50/100 μM	*—*	—		
Gypenoside	*In vivo*	Rat	MCT (55 mg/kg,s.c.)-induced PAH rats	Gypenosides 150 mg/kg/day	*i.g*	28 days	Reducing mPAP and RVSP, while inhibiting RVH and PVR.	Du et al. (2025)
18β-glycyrrhetinic acid	In vivo	Rat	MCT (60 mg/kg,s.c.)-induced PAH rats	*In vivo*:18β-GA 25/50/100 mg/kg/day	*—*	3 weeks	*In vivo*: Improvement of hemodynamic and histopathological parameters, decreases in the RVH, and alleviation of PVR.Inhibiting the proliferation of HPASMCs	Wang N. et al. (2022)
	In vitro	HPASMCs	PDGF-BB(20 ng/mL,24 h)-induced HPASMCs proliferation model	0–90 μM	*—*	48 h		
	In vivo	Rat	Rats were placed in an experimental chamber (simulating an altitude of 6,000 m) for 30 days	Hesperidin 25/50/100 mg/kg/day	*i.g*	15 weeks	Exerting protective effects against HAPH by comprehensively regulating the gut-lung axis	Fang et al. (2025)
	In vitro	Caco-2 cell	Hypoxia-induced Caco-2 cells	Hesperidin 8/16/32 μmol	*—*	—		
Hesperidin	In vivo	Rat	MCT (60 mg/kg,s.c.)-induced PAH rats	Hesperidin 20/40 mg/kg	*i.g*	14 days	It reduces RVSP, the fulton index, and mPAP. Concurrently, it improves pulmonary artery velocity-time integral and acceleration time, whilst mitigating pulmonary artery and right ventricular remodeling	He and Liao (2025)
Andrographolide	*In vivo*	C57BL/6J	Mice were subjected to either chronic hypoxia alone or a combined chronic hypoxia with i.p. injection of semaxanib	Andrographolide 1 mg/kg/day	*i.p*	4 weeks	Reducing distal PA remodeling, mPAP, and RVH.It also diminished the viability, proliferation, and migration of PASMCs isolated from PH patients, whilst increasing cellular apoptosis	Nie et al. (2021)
	*In vitro*	*HPASMCs*	Human PASMCs were isolated from the lung tissues of normal donors and patients with PH	ANDRO 1/3/10/30/60/100 μM	*—*	24 h		
Osthole	*In vivo*	Rat	MCT (50 mg/kg,s.c.)-induced PAH rats	10/20 mg/kg	*i.g*	28 days	Reducing mPAP and pulmonary weight index diminishes pulmonary artery wall thickening, decreases lumen enlargement, and alleviates interstitial inflammation	Li Y. et al. (2017)
Curcumin	*In vivo*	Rat	MCT (60 mg/kg)-induced PAH rats	50 mg/kg	*i.p*	7 days	Attenuating the development of RVH in a PAH rat model of myocardial ischaemia	Rice et al. (2016)
Betaine	*In vivo*	Rat	MCT-induced PAH rats	100/200/400 mg/kg/d	*i.g*	21 days	Improving abnormalities in RVSP, mPAP, RVH index, and pulmonary arterial remodeling induced by clonidine	Yang et al. (2018)
Resveratrol	*In vivo*	Rat	MCT (60 mg/kg,i.p.)-induced PAH rats	25 mg/kg	*i.g*	28 days	Inhibition of PASMC proliferation and pulmonary vascular remodeling	Shi et al. (2018C)
Astragalus polysaccharides	*In vivo*	Rat	MCT (60 mg/kg,s.c.) -Induced PAH rats	Astragalus polysaccharides 200 mg/kg once 2 days	*—*	2 weeks	Reduction in mPAP, decrease in PVR, reduction in RVH, and improvement in pulmonary artery remodeling	Yuan et al. (2017)

Till now, hesperidin is noteworthy, as a flavanone extracted from citrus fruits (Pyrzynska, 2022) with remarkable anti-inflammatory (Homayouni et al., 2018), antioxidant (Aalikhani et al., 2021), and cardioprotective activities (Pla-Pagà et al., 2019), and more interestingly, it could significantly suppress NF-κB and tumor necrosis factor-alpha (TNF-α) expression. Hesperidin would be a potential drug for the treatment of pulmonary arterial hypertension (Guan et al., 2025). Under normal physiological conditions, the NF-κB complex is complexed with the inhibitory protein IκB-α and remains inactive in the cytoplasm. Abnormal NF-κB activation results in the phosphorylation and degradation of IκB-α, ultimately leading to the activation of the retained NF-κB complex (primarily in the form of heterodimers consisting of p65 and p50 subunits). These complexes then translocate into the nucleus and upregulate the expression of pro-inflammatory and pro-angiogenic genes (Li et al., 2021). Hesperidin could inhibit the activation of the NF-κB pathway by attenuating CD68^+^ cell infiltration and further suppressing the expressions of p-p65 and p-IκB-α to relieve the pathological process of pulmonary hypertension, including pulmonary vascular remodeling, right ventricular remodeling, and pulmonary arterial endo-mesenchymal transition induced by MCT (He and Liao, 2025). Similar to hesperidin, gypenoside (Gyp) and osthole (Ost) also exhibit similar effects on the treatment of pulmonary hypertension. Gyp is a major metabolite of *Gynostemma pentaphyllum (Thunb.) Makino* (Lou et al., 2021). Gyp decreases P65 expression and inhibits NF-κB activation, downregulates the expression of proinflammatory genes IL-1β and IL-6, and attenuates PASMC proliferation (Du et al., 2025). Osthole, a natural coumarin-like metabolite isolated from Cnidium monnieri (L.) Cusson inhibits MCT-induced IκBα degradation and the subsequent activation of NF-κB p65 (Li Y. et al., 2017). In addition, Astragalus polysaccharide (APS), as the main active metabolite extracted from Astragalus mongholicus Bunge, could inhibit MCT-induced phosphorylation of IκBα and reduce the expression of various inflammatory markers and oxidants to decrease pulmonary vascular resistance and right ventricular hypertrophy (Yuan et al., 2017). Although the aforementioned natural products have demonstrated therapeutic efficacy against pulmonary arterial hypertension in animal models, and dose–response gradients have been used to define optimal dosing ranges, these studies are limited by reliance on a single disease model, which may not capture the heterogeneity observed in clinical practice.

Apart from the inhibition of p65 and IκB-α phosphorylation, hesperidin could also decrease the expression of occludin, Notch1, p-NF-κB/NF-κB, and TNF-α in intestinal tissues. In addition, hesperidin could also decrease the expression of proinflammatory cytokines IFN-γ, IL-1β, IL-6, and TNF-α in serum. Finally, by regulating the gut-lung axis (including gut microbiota rebalancing, intestinal anti-inflammation, choline metabolism regulation, and the normalization of pulmonary gene expression), it could protect against high-altitude pulmonary hypertension (Fang et al., 2025). Both preceding studies evaluating hesperidin for pulmonary hypertension were methodologically rigorous. In both *in vivo* and *in vitro* experiments, well-defined dose gradients were used to characterize pharmacologic effects and dose–response relationships. Moreover, the studies employed not only the widely used monocrotaline (MCT) model for PH but also a hypoxia-induced PH model established using a high-altitude, low-oxygen exposure chamber. This design enabled the simulation of PH arising from distinct etiologies, thereby improving real-world relevance and strengthening the generalizability of the findings. Notably, Fang and colleagues integrated multi-omics analyses, including genomics and metabolomics, with *in vitro* validation, providing convergent evidence that hesperidin ameliorates PH by inhibiting the NF-κB signaling pathway.

Baicalin (BAI) and baicalein (BE) are naturally isolated from the Scutellaria baicalensis Georgi, a dicotyledonous plant belonging to the family of Lamiaceae, and have broad biological and pharmacological activities (Wang R. et al., 2024). Baicalin could suppress the NF-κB signaling pathway by multiple mechanisms. Down-regulating Toll-like receptor-mediated signaling pathways at the most upstream level of the NF-κB signaling pathway, BAI could alleviate the pathological lesions and inflammatory response in the lung (Fu Y. J. et al., 2021; Wan et al., 2014). Concurrently, numerous experiments demonstrate that baicalin can also directly inhibit NF-κB activation by significantly reducing the ratio of phosphorylated NF-κB-p65 to total NF-κB-p65 levels and increasing I-κB protein levels. This action blocks TNF-α-induced NF-κB nuclear translocation, thereby preventing the transcription factor from entering the cell nucleus to exert pro-inflammatory effects (Xue et al., 2021). In addition, it is also reported that baicalin downregulates NF-κB-mediated inflammatory cascades by suppressing proinflammatory cytokine expression (TNF-α, IL-1β, IL-6) and cell adhesion molecule expression (vascular cell adhesion molecule-1 and intercellular adhesion molecule-1) (Luan et al., 2015). Meanwhile, BAI could promote phosphorylation of AKT and eNOS, which would further mediate vasodilation and suppress inflammation. It exerts overall regulation and ameliorates MCT-induced PAH, pulmonary vascular remodeling, and tissue fibrosis by modulating AKT/ERK/NF-κB signaling pathway (Yan et al., 2019). Baicalin can modulate multiple nodes of the NF-κB signaling pathway, ranging from inhibiting pathway activation to reducing pro-inflammatory mediator expression and attenuating downstream inflammatory cascades. Furthermore, baicalin regulates NF-κB activity through ERK signaling and p65 phosphorylation, thereby mitigating pulmonary vascular remodeling and the development of pulmonary hypertension. These findings suggest that natural products rarely act through a single signaling axis; instead, they often exert multi-target effects across interconnected pathways. Accordingly, by modulating multiple signaling pathways, natural products may attenuate pulmonary hypertension progression.

As shown above, similar to BAI, BE could also alleviate pulmonary inflammatory response by inhibiting the activation of the NF-κB signaling pathway, maintaining BMPR2 expression, suppressing endothelial-mesenchymal transition, and then inhibiting the pathological process of pulmonary hypertension (Shi et al., 2018a; Shi et al., 2018b).

The active metabolite 18β-Glycyrrhetinic acid (18β-GA) from Glycyrrhiza glabra L. markedly inhibited PDGF-BB-induced proliferation and DNA synthesis in Human Pulmonary Artery Smooth Muscle Cells (HPASMCs). The main mechanism underlying the inhibitory effects of 18β-GA on HPASMC proliferation was the prevention of NF-κB nuclear translocation, the downregulation of proinflammatory factors, including TNF-α, IL-6, and MCP-1, in cells, endoplasmic reticulum stress (ERS)-induced inflammatory response, and the inhibition of PERK/eIF2α/NF-κB signaling pathways (Wang et al., 2022).

TNF-α induces the canonical NF-κB pathway. Upon binding to TNFR1, TNF-α assembles the core signaling complex through a mechanism independent of protein modification, resulting in a series of non-degradative ubiquitination events that ultimately lead to the recruitment and activation of the IκB kinase (IKK) complex, which is a part of the NF-κB signaling cascade (Guo et al., 2024). Curcumin, a polyphenol found in Curcuma longa L, has been found to possess the same effects as baicalin, previously mentioned, both being able to attenuate MCT-induced increases of TNF-α and IL-1β, reduce the expression of inflammatory mediators, and ameliorate inflammation-induced right ventricular hypertrophy (Rice et al., 2016; Xue et al., 2021). Curcumin’s poor aqueous solubility and low bioavailability have limited its clinical translation. Rice and colleagues formulated curcumin into nanoparticles to evaluate its therapeutic efficacy. However, a key limitation is the use of intraperitoneal injection, which limits clinical feasibility relative to oral or other clinically deployable routes. In future studies, inclusion of a control group receiving unformulated (free) curcumin would provide a more direct comparison and strengthen evidence for the advantages of the nanoparticle formulation.

Heat shock protein 70 (Hsp70) is highly conserved in the NF-κB signaling pathway. Grape seed proanthocyanidins (GSP) are naturally extracted from the seeds of grapes (Vitis vinifera L.). HSP70 expression was downregulated by GSP, and GSP also inhibited NF-κB signaling by reducing the expression of p-IκBα. Simultaneously, betaine can significantly downregulate the expression of NF-κB and TNF-α (Yang et al., 2018). Both betaine and GSP can downregulate the inflammatory mediators IL-1β, IL-1, IL-6, and TNF-α elevated by MCT effects. In this way, betaine and GSP alleviate inflammation, relieve vasoconstriction and vascular remodeling, and improve the pathological process of pulmonary vascular remodeling and pulmonary arterial hypertension (Chen et al., 2018; Chen F. et al., 2019). However, Chen and colleagues evaluated only a single GSP dose, precluding a detailed assessment of the dose–response relationship. Moreover, the study did not include a positive control (standard-of-care) group, which prevents evaluation of the comparative benefit of GSP relative to established therapies.

Pattern recognition receptors (including TLRs) on innate immune cells induce the activation of a signaling cascade successively. TLR4 is the first Toll-like receptor identified in mammals and is involved in the upstream activation of NF-κB (Chen M. et al., 2021). Andrographolide, a diterpenoid lactone isolated from Andrographis paniculata (Burm.f.) Wall. ex Nees, reverses pulmonary vascular remodeling through attenuation of TLR4/NF-κB pathway–mediated inflammation (Nie et al., 2021). Meanwhile, cinnamaldehyde—a natural organic metabolite in Cinnamomum verum J. Presl—reversed the upregulation of TLR4 and HIF-1α expression induced by hypoxia in animal models and significantly reduced the p-p65/p65 ratio. The activation of TLR4/NF-κB/HIF-1α is inhibited, and vascular remodeling as well as endothelial dysfunction is improved (Zhang et al., 2025). In conclusion, Nie and colleagues strengthened the robustness of their study by combining two established *in vivo* models and by isolating human pulmonary arterial smooth muscle cells (PASMCs) from lung tissue for *in vitro* experiments. They further investigated the role of ANDR in PASMC proliferation, migration, and apoptosis. Similarly, Wang and colleagues evaluated 18β-glycyrrhetinic acid (18β-GA) in human PASMCs *in vitro*. However, both studies were largely confined to cell-based assays and therefore did not account for *in vivo* pharmacokinetics and metabolism that occur after administration under clinical conditions. Consequently, neither approach fully captures the complexity of human disease *in vivo*, which limits translational interpretability. To more comprehensively evaluate the efficacy of these natural products, future work should incorporate well-designed clinical trials to validate their therapeutic effects and safety in humans.

S1P is the catalytic product of SphK1 and can directly interact with the N-terminal RING domain of tumor necrosis factor receptor-associated factor 2 (TRAF2). Resveratrol is a natural polyphenolic compound with Reynoutria japonica Houtt. being its principal plant source (Zhao et al., 2023). Studies have shown that resveratrol inhibits SphK1, blocking the activation of the SphK1 (sphingomyelin kinase 1)/S1P (sphingomyelin 1-phosphate) signaling pathway from activating the NF-κB pathway. This subsequently suppresses the expression of the key factor cyclin D1—a primary regulator driven by NF-κB that promotes pulmonary arterial smooth muscle cell proliferation. Ultimately, suppressing pathological vascular remodeling and reducing pulmonary arterial pressure to treat PAH (Shi et al., 2018c). This study used specific inhibitors to delineate the molecular target(s) of resveratrol, showing that it attenuates monocrotaline (MCT)-induced pulmonary arterial remodeling by inhibiting the SphK1/NF-κB signaling pathway. These findings provide supportive mechanistic evidence that resveratrol may represent a potential therapeutic candidate for pulmonary arterial hypertension. However, the study evaluated resveratrol at only a single concentration, which limits interpretation of its therapeutic profile. The absence of dose-ranging experiments precludes assessment of the dose–response relationship and prevents determination of the optimal therapeutic dose and its impact on efficacy.

### 4.2 PI3K/AKT signaling pathway

**FIGURE 4:**
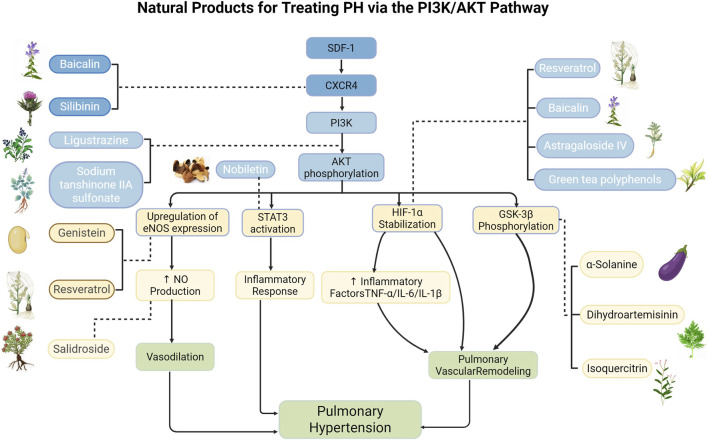
Natural products for treating PH via the PI3K/AKT pathway (created with BioRender-QG295TXB7L).

The phosphoinositide 3-kinase/protein kinase B (PI3K/AKT) signaling pathway is an essential component of the intracellular signaling network that positively regulates the inflammatory response. On one hand, it can promote the onset of inflammation. On the other hand, it also participates in inflammation resolution and tissue repair (He X. et al., 2022; Wu et al., 2023). In endothelial cells, activated AKT promotes the phosphorylation of eNOS, thereby increasing NO production and exerting a vasodilatory effect (Yao et al., 2013). Pulmonary vascular remodeling (PVSR), a typical pathological manifestation in end-stage PH, proceeds in a close relationship with pathological proliferation of PASMCs (Abudukeremu et al., 2025) (Figure 4) (Table 2).

**TABLE 2:** Natural products acting via the PI3K/AKT signaling pathway.

Natural products acting via the PI3K/AKT signalling pathway								
Natural products	Model used	Species	Model’s induction method	Intervention doses	Administration	Periods	Efficacy	References
Nobiletin	*In vivo*	Rat	MCT-induced PAH rats;	Nobiletin 1/5/10 mg/kg	*i.g*	3 weeks	Reducing mPAP and PVR improves RVH and pulmonary arterial remodeling, and inhibits the proliferation of PASMCs	Yin et al. (2023)
	*In vitro*	PASMCs	PASMCs induced by PDGF-BB(20 ng/mL)	10 μmol/L	*—*	—		
Sodium tanshinone II sulfonate A	*In vivo*	Rat	Hypoxic (in the hypoxic chamber for 8 h per day for 3 weeks) PH rats	STS 30 mg/kg	*i.p*	3 weeks	Reducing PAP, improving RVH, pulmonary oedema, and pulmonary vascular proliferation	Bao et al. (2020)
Baicalin	*In vivo*	Mice	Hypoxic (8 h per day,28 days) PH mices	Baicalin 60 mg/kg	*i.p*	28 days	Improving haemodynamic changes, RVH, pulmonary congestion, and alleviating hypoxia-induced pulmonary arterial remodeling and morphological alterations	Huang et al. (2017)
	*In vivo*	Rat	Hypoxic PH rats	Baicalin 100 mg/kg	*i.p*	14 days	Repressing the elevation of RVSP, RV/LV + S, and attenuating the pulmonary vascular structure remodeling (PVSR) of pulmonary arterioles induced by chronic hypoxia. Suppress the proliferation of PASMCs	Zhang et al. (2014)
	*In vitro*	PASMCs	Hypoxia-induced PASMCs	Baicalin 10/20 μmol/L	*—*	—		
Resveratrol	*In vivo*	Rat	Hypoxic PH rats	Resveratrol 40 mg/kg/day	*i.g*	28 days	Alleviating hypoxia-induced RVSP and pulmonary arterial remodeling.Inhibiting the proliferation of PASMCs	Xu et al. (2016)
	*In vitro*	PASMCs	Hypoxia-induced PASMCs	Resveratrol 10/20/40 μmol/L	*—*	—	​	​
	*In vitro*	PASMCs	Hypoxia-induced human PASMCs	Resveratrol 40/80/100 μM	*—*	—	Preventing HPASMC proliferation	Chen et al. (2014)
	*In vitro*	PASMCs	Hypoxia-induced human PASMCs	Resveratrol 10/30/100 μmol/L	*—*	—	Inhibition of PASMC proliferation and migration	Guan et al. (2017)
Ligustrazine	*In vivo*	Rat	MCT (60 mg/kg,s.c.)-induced PH rats	Ligustrazine 40/80/160 mg/kg/day	*—*	4 weeks	Alleviating MCT-induced RVSP, RVH, and PVR.Inhibiting the proliferation and inflammation of PASMCs	Huang et al. (2021)
	*In vitro*	PASMCs	PASMCs induced by PDGF-BB	—	*—*	—		
α-Solanine	*In vivo*	Mice	MCT (60 μg/kg,i.p.)-induced PAH C57BL/6J mice	5 μg/g	*i.p*	2vweeks	Reducing distal pulmonary artery remodeling, mean pulmonary artery pressure, and right ventricular hypertrophy	Nie et al. (2017)
	*In vitro*	PASMCs	Hypoxia (2.5% O2, 4.5% CO2 and 92% N2)-induced PASMCs	6/8/10/12/14 μmol/L	*—*	48 h		
Isoquercitrin	*In vivo*	Rat	MCT (40 mg/kg)-induced PAH rats;	0.1% IQC maintain feed	*—*	3 weeks	Reducing right ventricular systolic pressure (RVSP), the RV/LV + S ratio, and RVH, and improving pulmonary vascular remodeling	Zhang et al. (2017)
	*In vitro*	PASMCs	PASMCs induced by PDGF-BB(20 ng/mL)					
Genistein	*In vivo*	Rat	Hypoxic (21 days) PH rats	60 mg/kg	*i.g*	3 weeks	Hypoxic pulmonary hypertension may be alleviated by correcting its chronic structural remodeling component and its pulmonary abnormal vasoconstriction component	Kuriyama et al. (2014)
	*In vitro*	HUVECs	Hypoxia-induced HUVECs	Gen 10 μM	*—*	48 h		
	*In vitro*	HepG2 cells	HepG2 cells under hypoxia	Gen 10 μM	*—*	48 h		
Astragaloside IV	*In vivo*	Rat	MCT (60 mg/kg,i.p.)-induced PAH rats	ASIV 10/30 mg/kg	*i.p*	21 days	Improving the pathological changes in pulmonary artery structural remodeling, pulmonary arterial pressure, and right ventricular hypertrophy in a rat model of MCT PAH.	Jin et al. (2021)
	*In vitro*	HPASMCs	Hypoxia-induced HPASMCs	ASIV 10/20/40/80 μM	*—*	24 h		
Dihydroartemisinin	*In vivo*	*rat*	MCT (50 mg/kg,i.p.)-induced PAH rats	DHA 50/100 mg/kg	*i.g*	3 weeks	DHA inhibited platelet-derived growth factor-BB-mediated pulmonary arterial smooth muscle cells proliferation and migration in a dose-dependent manner. And DHA inhibited pulmonary vascular remodeling	Tang et al. (2020)
	*In vitro*	*PASMCs*	PASMCs induced by PDGF-BB(20 ng/mL)	DHA 5/10/20/40 mM	*—*	24 h		

Targeting the inhibition of pulmonary arterial smooth muscle cell (PASMCs) abnormal proliferation as a novel therapy for PH is of great significance in the clinic. Genistein (Gen), a phytoestrogen extracted from Glycine max (L.) Merr, can treat vasoconstriction and chronic structural remodeling by restoring NO-mediated signaling through PI3K/Akt regulation, thereby attenuating hypoxia-induced PH (Kuriyama et al., 2014). This study reports initial evidence that Gen ameliorates pulmonary hypertension via the EPO/EPOR system and the PI3K/Akt pathway, supported by both *in vivo* experiments and *in vitro* validation in the human hepatocyte line HepG2 and human umbilical vein endothelial cells (HUVECs). However, the study lacked dose-ranging control groups, limiting assessment of the dose–response relationship and hindering determination of an optimal dosing range.

The cell cycle is strictly regulated by cyclin-dependent kinases (CDKs) and CDK inhibitors, maintaining the balance between cell proliferation and cell quiescence (Chen et al., 2024). P27 is a CDK inhibitor that can directly interfere with the G1-S transition by inhibiting the G1-phase CDK/cyclin complexes and further suppresses cell proliferation (Zhang H. et al., 2024). G0/G1-to-S transition is a key switch in the cell cycle to proceed from quiescence/G1 phase to DNA synthesis (S phase). The imbalance of G0/G1-to-S transition leads to uncontrolled cell proliferation (Adu-Amankwaah et al., 2025). Ligustrazine, an alkaloid monomer isolated from Conioselinum anthriscoides ‘Chuanxiong', can block G0/G1-to-S transition by modulating the PI3K/AKT signaling pathway, further inhibiting proliferation and inflammation in PASMCs (Huang et al., 2021). This study integrated *in vivo* and *in vitro* experiments and employed multiple dosing regimens, enabling assessment of dose-dependent effects. This design strengthens the robustness of the findings and facilitates the identification of the optimal therapeutic dose.

Baicalin reduces hypoxia-inducible factor-1α (HIF-1α) production through the AKT signaling pathway and protects p27 degradation. This may ameliorate chronic hypoxia-induced PVR and PAH (Zhang et al., 2014). HIF-1α is critically involved in oxygen homeostasis and in hypoxia-induced vascular smooth muscle remodeling and development of PH (Liu et al., 2022). Astragaloside IV is a purified low-molecular-weight saponin contained in Astragalus mongholicus Bunge. Research suggests that astragaloside IV downregulates HIF-1α protein expression in HPASMCs and can also inhibit hypoxia-induced TNF-α and IL-1β release in HPASMCs. These results suggest that astragaloside IV can improve inflammation, pulmonary endothelial cell dysfunction, PASMC proliferation, and resistance to apoptosis, and attenuate MCT-induced PAH (Jin et al., 2021). Similar to astragaloside IV, green tea polyphenols could also inhibit the proliferation and migration of PASMCs by suppressing HIF-1α and reducing AKT phosphorylation expression (Yang et al., 2024). Compared with the other two studies, Yang and colleagues primarily relied on network pharmacology, predicting multiple targets and pathways to infer the potential therapeutic effects of tea polyphenols. However, the experimental component was confined to *in vitro* assays, without *in vivo* models or clinical validation, which limits the strength of the empirical evidence. Furthermore, the study did not account for *in vivo* pharmacokinetics and metabolism following administration, thereby limiting the translational relevance of the findings. Consequently, although this work provides a useful theoretical framework, the results should be interpreted as hypothesis-generating and preliminary. Additional *in vivo* and/or clinical studies are needed to validate efficacy and elucidate the underlying mechanisms.

Similarly, resveratrol can also inhibit hypoxia-induced proliferation of HPASMCs by suppressing the PI3K/AKT pathway through either reducing HIF-1α activity or decreasing AKT phosphorylation (Guan et al., 2017). In addition, resveratrol could inhibit inflammation induced by chemokines and cytokines induced by TGF-β, PDGF-α, PDGF-β, IL-1β, IL-18, IL-8, IL-10, and especially IL-6, which were mediated by resveratrol, and thereby ameliorate PAH (Chen et al., 2014; Mirhadi et al., 2021; Xu et al., 2016). Similar to animal models and cell experiments, network pharmacology analysis also confirms that resveratrol could treat PAH via activating the PI3K/AKT signaling pathway (Chen et al., 2020). In 2020, Chen and colleagues used network pharmacology to predict potential targets for resveratrol in PH, which may provide a useful reference. However, the experiments by Guan and colleagues and Bernadette Chen and colleagues were confined to *in vitro* cellular systems, without validation in animal models. Although Xu and colleagues incorporated both *in vivo* and *in vitro* studies, the work did not include head-to-head comparisons with standard-of-care therapies, which limits assessment of translational potential.

The upstream pathway of PI3K/AKT is SDF-1/CXCR4. SDF-1/CXCR4 plays a crucial role in vascular remodeling. Chemokine stromal cell-derived factor-1 (SDF-1), a member of the C-X-C chemokine subfamily, mediates its functional effects through its receptor, chemokine receptor 4 (CXCR4) (Liu et al., 2025). Baicalin attenuates HPH by inhibiting SDF-1XCR4-induced PI3K/AKT via activation of A2A receptor (A2AR), which is involved in inflammatory and immune responses (Huang et al., 2017). In contrast with baicalin, silibinin, a polyphenolic flavonoid extracted from Silybum marianum (L.) Gaertn, can upregulate the gene expression of inflammatory markers IL-6 and TNFα by inhibiting the CXCR4/SDF-1 pathway to reduce pulmonary arterial pressure and pulmonary arteriolar occlusion and ameliorate pulmonary vascular remodeling (Zhang et al., 2020; Zhang T. et al., 2019).

Signal Transducer and Activator of Transcription 3 (STAT3) is not only regulated by PI3K, but also reciprocally activates the PI3K/AKT pathway (Banerjee and Resat, 2016; Liang et al., 2019). Nobiletin Inhibits PDGF-BB-Induced Pulmonary Arterial Smooth Muscle Cell Proliferation and Inflammation via PI3K/Akt/STAT3 Signaling Pathway. Attenuates MCT-Induced Rat PAH via PI3K/Akt/STAT3 Signaling Pathway (Yin et al., 2023). Different from nobiletin, as the main active metabolite of Salvia miltiorrhiza Bunge, Sodium tanshinone II sulfonate A directly inhibits the PI3K/AKT/mTOR signaling pathway, arrests hypoxia-induced vascular remodeling and fibrosis. simultaneously greatly decreases the contents of proinflammatory factors such as IL-6, IL-8, and TNF-α, and exerts anti-inflammatory action by suppressing inflammatory response in SD rats, and ultimately alleviates the development process of PAH (Bao et al., 2020).

Glycogen synthase kinase 3β (GSK3β) is a serine/threonine kinase. GSK-3β acts as a downstream target of the PI3K/Akt signaling pathway (Hermida et al., 2017), and its activity is modulated by Akt phosphorylation. GSK3β may regulate many subsequent cascade reactions and participate in the vascular remodeling process in PAH (Sklepkiewicz et al., 2011). α-Solanine, dihydroartemisinin (DHA), and isoquercitrin can ameliorate PAH through modulating the vascular remodeling and inflammation by improving the GSK-3 signaling pathway to different targets. α-Solanine is primarily found in the tuber of potato (Solanum tuberosum L.), whereas isoquercitrin is mainly a plant metabolite of Betula pendula subsp. mandshurica (Regel) Ashburner and McAll (Ning et al., 2022; Xiang et al., 2022). Although the three botanical drugs have slight differences in regulating GSK-3, the two botanical drugs, α-Solanine and Isorhamnetin, mainly inhibit GSK-3 activity by suppressing Akt/GSK-3 phosphorylation, while Dihydroartemisinin enhances GSK-3β-mediated β-catenin degradation by upregulating GSK-3β expression. Specifically, α-Solanine mainly suppressed the abnormal proliferation and angiogenesis in pulmonary arterial endothelial cells by inhibiting the activation of Akt/GSK-3α, especially the phosphorylation of GSK-3α at Ser21 (Nie et al., 2017). Dihydroartemisinin is an important derivative of Artemisinin, a natural product isolated from the Chinese botanical drug Artemisia annua L. DHA enhanced the inhibition of Wnt/β-catenin signaling by upregulating GSK-3β levels and promoting the activity of GSK-3β. This promoted the complex formation between AXIN2 and other proteins, accelerated the degradation of β-catenin, and finally reduced the proliferation and migration of PASMC (Tang et al., 2020). Isoquercitrin inhibited the phosphorylation of Akt/GSK3β induced by PDGF-BB, and affected the expression of its downstream target Cyclin D1 and CDK4. Meanwhile, isoquercitrin also prevented the degradation of p27Kip1, leading to cell cycle arrest of PASMC and suppression of cell proliferation (Zhang et al., 2017). The aforementioned studies predominantly employed integrated *in vivo* animal models and *in vitro* cellular assays to demonstrate efficacy, thereby strengthening the robustness of the findings. However, although each study examined mechanisms of action, dose-ranging analyses and systematic evaluation of potential adverse effects were limited. Consequently, a comprehensive assessment of clinical efficacy and safety was not performed. Future research should prioritize clinical translatability, including well-designed dose-optimization studies and safety assessments, to support eventual clinical application.

### 4.3 PPARγ signaling pathway

PPARγ is a master regulator in the inflammation-resolution balance. Peroxisome proliferator-activated receptor gamma (PPARgamma) is a ligand-activated transcription factor in the nuclear receptor superfamily that regulates glucose and lipid metabolism, endothelial function, and vascular inflammation (Pawlak et al., 2015; Rabinovitch et al., 2014) (Table 3).

**TABLE 3:** Natural products acting via the PPARγ signaling pathway.

Natural products acting via the PPARγ signalling pathway								
Natural products	Model used	Species	Model’s induction method	Intervention doses	Administration	Periods	Efficacy	References
Aloperine	*In vivo*	Rat	MCT (50 mg/kg,i.p.)-induced PAH rats	Aloperine 50/100 mg/kg/day	*i.g*	2 weeks	Inhibiting RVSP and RVH, normalising PA remodeling, improving right ventricular function as measured by transthoracic echocardiography indices, and suppressing excessive proliferation of pulmonary arteries and PASMCs	Shan et al. (2023)
	*In vitro*	PASMCs	Primary rat distal PASMCs were cultured using enzymatic methods	Aloperine 25/50/100 μM	*—*	—		
Grape seed procyanidin	*In vivo*	Rat	Rats exposed to cigarette smoke	30 mg,2 mL/kg	*i.t*	6 months	Reversing pulmonary vascular remodeling	Liu J. et al. (2020)
	*In vitro*	PASMCs	Rat primary PASMCs were isolated from peripheral small pulmonary arteries	4 g/L	*—*	24 h		
Puerarin-V	*In vivo*	Rat	MCT (50 mg/kg,sco)-induced PAH rats	Puerarin-V 10/30/100 mg/kg/day	*i.g*	28 days	Reducing RVSP and pulmonary injury, improving pulmonary arterial blood flow and pulmonary vasodilatory and contractile function, mitigating right ventricular injury and remodeling, and maintaining normal right ventricular function	Chen et al. (2022)
	*In vivo*	Mice	Hypoxia (10%O2)-induced PH mice	Puerarin-V 60 mg/kg/day	*i.g*	7days		
	*In vitro*	HPASMCs	Hypoxia-induced HPASMCs	Puerarin-V 3/10/30 μM	*—*	48 h		
Sodium tanshinone IIA sulfonate	*In vivo*	Rat	Hypoxia-induced PAH rats	30 mg/kg	*i.p*	21 days	Reducing hypoxia-induced increase of mean right ventricular pressure, RVSP, and RV/(LV + S) and reversing hypoxia-induced pulmonary vascular remodeling	Jiang et al. (2016)
	*In vitro*	HPASMCs	Hypoxia-induced HPASMCs	—	*—*	—		

ET-1 is a key vasoactive mediator that promotes the sustained vasoconstriction and vascular remodeling phenotypes of PAH. Deletion of PPARγ in vascular smooth muscle cells (VSMCs) significantly enhanced the ET-1–induced vasoconstrictive responses as well as the associated oxidative stress, inflammatory activation, and structural remodeling, indicating that PPARγ plays a protective role in mediating vascular injury induced by ET-1 (Lago-Docampo et al., 2022). In addition, activation of PPARγ can increase NO bioavailability by promoting NO release and simultaneously attenuating the expression of pro-oxidant signaling cascades, which can relieve the pathophysiological process of pulmonary hypertension (Chanana et al., 2022; Ketsawatsomkron et al., 2010).

Genistein, extracted from Glycine max (L.) Merr, can not only alleviate hypoxic pulmonary arterial hypertension by enhancing nitric oxide signaling and the erythropoietin system, but is also regarded as a PPAR agonist. Network pharmacology studies revealed that there is a positive relationship between PPARγ and the “pulmonary hypertension” phenotype of potential targets of genistein, which is directly connected to the pathogenesis of pulmonary hypertension and further considered as a potential target of pulmonary hypertension (PH) (Chen Y. et al., 2019). PPARγ can promote the expression of PI3K, which in turn activates Akt and phosphorylates eNOS to produce NO. Puerarin extracted from the Pueraria montana var. lobata (Willd.) Maesen and S.M.Almeida ex Sanjappa & Predeep can improve the pulmonary artery and right ventricular remodeling through the PPARγ/PI3K/Akt/eNOS signaling pathway (Chen et al., 2022). This study employed both hypoxia-induced and MCT-induced PH models, enabling more comprehensive validation of efficacy across etiologically distinct PH conditions. This design provides stronger support for the drug’s broader applicability and translational potential.

KCNQ potassium channels are a large subclass of voltage-gated potassium channels (Kv), and there are five subtypes, from KCNQ1 to KCNQ5. KCNQ5 activation causes cellular hyperpolarization and mediates sGC stimulator-induced pulmonary arterial vasodilation in PASMCs (Mondéjar-Parreño et al., 2019). Endogenous NO diffuses out of the cell and binds and activates sGC, whereupon sGC catalyses the conversion of guanosine triphosphate (GTP) to cyclic guanosine monophosphate (cGMP), and further activates cGMP-dependent protein kinase (PKG) to mediate subsequent downstream signaling and induce vasodilation and antiproliferation (Zuccarello et al., 2020). It has been reported that the active vasodilator aloperin (ALO) from the seeds and leaves of the herbal plant Sophora alopecuroides L. can activate KCNQ5 channels and induce the cGMP/PKG/PPARγ signaling pathway and can then inhibit PASMC proliferation (Shan et al., 2023). Interestingly, the active metabolite danshinsu IIA sodium sulfonate (STS), a metabolite of the Chinese botanical drug Danshen (Salvia miltiorrhiza Bunge), can prevent hypoxia-induced increases in intracellular calcium homeostasis and cell proliferation by targeting and restoring the hypoxia-inhibited PKG-PPAR-γ signaling pathway in PASMCs (Jiang et al., 2016).

Cyclooxygenase-2 (COX-2) is also called prostaglandin-endoperoxide synthase (PTGS). It is an inflammatory mediator that participates in the inflammatory process (Wu N. et al., 2022). As a catalytic enzyme, COX-2 promotes inflammatory response by converting arachidonic acid into other typical pro-inflammatory substances, such as TXA_2_, PGF_2_α, and PGE_2_ (Lacy et al., 2016). Upon binding to its ligand and translocating to the nucleus, PPAR-γ suppresses COX-2 expression, thereby alleviating inflammation (Xiao et al., 2021). Bioactive polyphenolic flavonoid complexes extracted from grape seeds (Vitis vinifera L.), known as GSP, not only improve pulmonary hypertension (PH) by modulating the NF-κB pathway, but also exert beneficial effects through the PPARγ pathway. GSP reverses pulmonary vascular remodeling by modulating PPAR-γ/COX-2 pathway to treat inflammation in cigarette smoke-induced PAH (Liu J. et al., 2020). Liu and colleagues did not use the conventional monocrotaline (MCT) model; instead, they employed a cigarette smoke–induced PH model to simulate PH arising from an alternative clinical etiology.

### 4.4 MAPK signaling pathway

The MAPK pathway is a three-tiered cascade signaling network consisting of protein phosphorylation. It is involved in the regulation of cell proliferation, differentiation, apoptosis, as well as inflammatory and immune responses. It is one of the major inflammation-related signaling pathways in the body. The family contains members such as ERK, p38MAPK, JNK, etc (Guo et al., 2020). The MAPK pathway is activated and then induces the expression of downstream target genes, leading to the production of pro-inflammatory cytokines such as interleukin-6 (IL-6), interleukin-8 (IL-8), tumor necrosis factor-α (TNF-α), and other inflammatory mediators, causing inflammatory reactions. Toll-like receptors (TLRs) can induce MAPK activation. TLRs can activate MAPK in a synergistic manner with the NF-κB pathway to translate extracellular signals into cellular responses (Zhao et al., 2017). Among them, p38- MAPK (p38 mitogen-activated protein kinase) is a member of the MAPK signaling family. It plays a central regulatory role in initiating, amplifying, and sustaining inflammatory responses. Clinical application of FDG-PET imaging showed that p38 mitogen-activated protein kinase (p38MAPK) inhibitor BMS-582949 attenuates inflammatory responses in atherosclerotic plaques. Animal model studies have also demonstrated that p38αMAPK signaling can exhibit pro-inflammatory or anti-inflammatory effects (Raza et al., 2017) (Table 4). P38-MAPK is a new target for PAH. Research indicates that inhibiting p38 MAPK prevents the differentiation of fibroblasts into myofibroblasts induced by transforming growth factor beta-1 (TGF-β1) (Chang et al., 2022). In addition, animal model studies on pulmonary hypertension have also shown that inhibiting p38 MAPK activity can prevent and reverse pulmonary vascular remodeling (Church et al., 2015).

**TABLE 4:** Natural products acting via the MAPK signaling pathway.

Natural products acting via the MAPK signalling pathway								
Natural products	Model used	Species	Model’s induction method	Intervention doses	Administration	Periods	Efficacy	References
Resveratrol	*In vivo*	Rat	Acute PTE-induced PH	10 mg/kg/day	*i.p*	—	Reducing mPAP.	Chun et al. (2012)
Magnesium lithospermate B	*In vivo*	Mice	Pulmonary artery banding (PAB) model	MLB 10 mg/kg/day	*i.p*	4 weeks	Offering the therapeutic potential for the patients of RV dysfunction	Qu et al. (2020)
	*In* *vitro*	The rat alveolar macrophage NR8383 cell	The macrophages were stimulated by SP separately	MLB 10/25/50 μM	*—*	24 h		
	*In vitro*	Fibroblasts	The fibroblasts were stimulated by SP separately	MLB 25 μM	*—*	1 h		
Astragaloside IV	*In vivo*	Rat	MCT-induced PAH rats	10/30 mg/kg	*i.p*	21 days	Improving pulmonary endothelial cell dysfunction, abnormal proliferation, and apoptosis of pulmonary arterial cells	XinTian et al. (2022)
	*In vitro*	HPASMCs	Hypoxia-induced HPASMCs	—	*—*	48 h		
Mimosa pigra	*In vivo*	Rat	Hypoxia-induced PH rats	Mimosa pigra 50 mg/kg/d	*i.p*	21 days	Improving haemodynamic parameters and right ventricular hypertrophy	Rakotomalala et al. (2013)
	*In vitro*	Human vascular endothelial cells	TNFα-induced inflammatory model in human vascular endothelial cells	—	*—*	—		
Capsaicin	*In vivo*	Rat	MCT (60 mg/kg,i.p.)-induced PAH rats	Capsaicin 1 mg/kg/d	*i.p*	28 days	Reducing RVSP, RV/(LV + S), RV/BW, LW/BW.	Xu et al. (2017)
	*In* *vitro*	NK8383 cells	LPS, 10 ng/mL or substance P,1 μg/mL induced NK8383 cells	—	*—*	—		
Ginsenoside	*In vivo*	Rat	MCT (60 mg/kg,i.p.)-induced PAH rats	Ginsenosides 20/40/80 mg/kg/d	*i.p*	18 days	Reducing RVSP, RVHI, and LW/BW. Improving pulmonary vascular remodeling	Qin et al. (2016)
Paeonol	*In vivo*	Mice	Hypoxia-induced PH mice	Paeonol120 mg/kg/d	*i.g*	10 days	Improving right ventricular function and alleviating hypoxia-induced proliferation of pulmonary arterial smooth muscle cells	Zhang L. et al. (2018)
	*In vitro*	HPASMCs	Hypoxia-induced HPASMCs	Paeonol 100/200 μmol/L	*—*	—		

The extract from Mimosa pudica L. exerts effects similar to those of p38 MAPK inhibitors. MPG attenuates p38 MAPK activation in hypoxic rats to exert antioxidant and anti-inflammatory effects and induces endothelium-dependent vasorelaxation by stimulating NO production (Rakotomalala et al., 2013). However, this study did not identify the specific plant metabolites that were active in the experiments. Further research is needed to explore and compare the effects of different Mimosa pudica L. extracts in disease contexts. Similarly, the magnesium salt form of Salvia miltiorrhiza Bunge, Magnesium lithospermate B (MLB), could alleviate inflammation and improve right ventricular remodeling by attenuating phosphorylated p38 (p-p38) MAPK, pulmonary artery banding (PAB)-induced macrophage inflammatory cytokines IL-1β and IL-6 expression, and increasing TNF-α (Qu et al., 2020). In animal experiments using an MCT-induced PAH model, intervention with capsaicin—the main pungent metabolite in Capsicum annuum L—produced similar results. Meanwhile, in the rat alveolar macrophage cell line NR8383, intervention with capsaicin pretreatment inhibited the p38MAPK pathway to attenuate the inflammatory response in PAH (Xu et al., 2017). Both the aforementioned MLB and capsaicin studies employed a prophylactic dosing regimen, with capsaicin administered 3 days before MCT injection and MLB dosing initiated at the time of model induction. Although this design may facilitate mechanistic interrogation, it diverges from clinical practice because patients typically receive treatment after PAH onset. In addition, the animal models differed fundamentally: the MCT model primarily reflects inflammation-driven pulmonary vascular injury, whereas the pulmonary artery banding (PAB) model represents isolated right ventricular pressure overload and does not involve primary pathological alterations in the pulmonary vasculature. This heterogeneity in model selection complicates the interpretation of whether p38MAPK inhibition acts directly on the pulmonary vasculature or primarily modulates right ventricular remodeling and function.

Monocyte chemotactic protein-1 (MCP-1) promotes monocyte recruitment, induces cytokine production, participates in inflammatory positive feedback caused by acute pulmonary vascular injury, and acts as a key mediator that induces inflammatory cell infiltration into the lungs (Lin et al., 2019). An acute pulmonary thromboembolism (PTE) model was established by infusing autologous blood clots into pulmonary arteries via polyethylene catheters. In the early stages of the rat PTE model, MCP-1 levels significantly increased around pulmonary artery walls (Lin et al., 2019). Chun’s study has reported that resveratrol could alleviate acute PTE-induced PH by inhibiting the activation of p38MAPK and expression of MCP-1 (Chun et al., 2012). The study’s strength lies in the use of an autologous thrombus model that more closely approximates clinical conditions, together with pathway validation using specific inhibitors.

As described in previous sections, we have talked about how impaired NO production may induce pulmonary hypertension, and furthermore, daily administration of NO donors can reverse pulmonary hypertension and pulmonary vascular remodeling induced by MCT (Hampl and Herget, 2000). Ginsenosides, the main active metabolites in Panax ginseng C.A.Mey, can upregulate the MKP-1 expression and downregulate the expression of phosphorylated proteins p-ERK, p-p38MAPK, and p-JNK1/2 in the MAPK pathway. Ginsenosides inhibit the MAPKs signaling pathway to act on pulmonary vascular remodeling. Meanwhile, it can improve MCT-induced pulmonary hypertension pathology in rats by upregulating eNOS protein expression and promoting NO and cGMP formation (Qin et al., 2016). This study used ginsenoside extracts rather than purified individual metabolites. Ginseng contains dozens of ginsenoside monomers, and their pharmacological effects may be synergistic or antagonistic. Consequently, the use of whole extracts makes it difficult to unambiguously identify the principal active metabolites and quantify their respective contributions.

ERK1/2 represent the final two components in the mitogen-activated protein kinase (MAPK) phosphorylation cascade and are essential modules in multiple signaling pathways regulating cell behavior and fate (Lucas et al., 2022). Paeonol exerts therapeutic effects on PAH by targeting the ERK1/2 signaling pathway. It modulates the phosphorylation levels of ERK1/2 and shortens the DNA replication phase (S phase) and mitotic phase (M phase) of the cell cycle, thereby inducing growth inhibition and cell cycle arrest (Zhang L. et al., 2018). Different from paeonol, Astragaloside IV (ASIV), the main active metabolite from Astragalus mongholicus Bunge, can not only decrease the expression of p-ERK1/2 protein, but also upregulate the expression of Bax, cleaved caspase-9, and cleaved caspase-3, and downregulate the expression of Bcl-2 in HPASMCs. Finally, ASIV can decrease PASMC proliferation and apoptosis resistance (XinTian et al., 2022). Among these, Zhang et al. focused on the regulation of the ERK1/2-cyclin axis, presenting a relatively complete mechanism chain. However, their research depth was limited, as they did not employ specific inhibitors or genetic intervention methods for validation. In contrast, integrated analyses encompassed multiple levels of indicators including proliferation, apoptosis, and inflammation, yielding rich information. Yet, the causal relationships between the various mechanisms remain insufficiently clear.

### 4.5 JAK/STAT signaling pathway

JAK-STAT signaling pathway (Janus kinase–signal transduction and transcription activation factor pathway), also called the IL-6 signaling pathway, is a cytokine stimulatory signal transduction pathway for transmitting signals from cells to their microenvironment (Peng et al., 2023). It is composed of three components: tyrosine kinase-related receptors, Janus kinases (JAKs), and transcription factors STAT. When cytokines such as IL-6, TNF-α, IL-1β, or growth factors bind to JAK receptors, they dimerize. Subsequently, the activated JAKs phosphorylate certain tyrosine residues of STAT proteins. Phosphorylated STAT dimers translocate into the nucleus and induce changes in DNA transcription, allowing efficient signal transmission from the extracellular space to the nucleus (Hu et al., 2021). The JAK-STAT pathway is involved in many physiological and pathophysiological processes. Its abnormal activation is associated with the occurrence and development of many diseases, including autoimmune diseases, and it is widely involved in cell proliferation, differentiation, apoptosis, metabolism, immune regulation, inflammation, and the maintenance of hematopoiesis (Philips et al., 2022) (Table 5).

**TABLE 5:** Natural products acting via the JAK/STAT signaling pathway.

Natural products acting via the JAK/STAT signalling pathway								
Natural products	Model used	Species	Model’s induction method	Intervention doses	Administration	Periods	Efficacy	References
Magnolol	*In vivo*	Rat	Hypoxia (in a hypoxic chamber,10% O2)-induced PH rats	Magnolol 10/20 mg/kg/d	*i.g*	4 weeks	Attenuating RV hypertrophy and fibrosis	Fu M. et al. (2021)
	*In vitro*	H9C2	Hypoxia-induced H9C2	Magnolol 10/20 μM	*—*	—		
Ginsenoside Rg1	*In vivo*	Mice	Hypoxia (10%O2 4weeks)-induced PH mice	5/10/20 mg/kg/d	*i.g*	4 weeks	Alleviating the abnormal elevation of mPAP, RVSP and RVHI, and improving pulmonary vascular remodeling	Ran et al. (2024)
	*In vitro*	PASMCs	Hypoxia-induced PASMCs	Rg1 20 μM	*—*	—		
Dihydromyricetin	*In vivo*	Rat	MCT (60 mg/kg,i.p.)-induced PAH rats	DHM 100 mg/kg	*i.g*	28 days	Improving abnormal changes in RVSP, RVHI, PAR, and inhibiting HPASMC migration	Li Q. et al. (2017)
	*In vitro*	HPASMCs	IL-6-induced HPASMCs	DHM 10/50/100 μM	*—*	12 h		

Matrix metalloproteinase-9 (MMP9) is one of the most extensively studied enzymes in the MMP family. MMP9 directly degrades ECM proteins such as collagen and elastin and is involved in pulmonary vascular remodeling (Kalali, 2023; Vandooren et al., 2013). Phosphorylated STAT3 (p-STAT3) recruits transcription coactivators to induce the transcription of the MMP9 gene and further regulate the expression of MMP9 (Ghosh et al., 2015). Dihydromyricetin (DHM), a flavonoid extracted from Hovenia dulcis Thunb, exhibited similar effects to STAT3 inhibitors. In animal and cellular experiments, DHM inhibited the expression of PY-STAT3 and MMP9 protein induced by IL-6 as well as HPASMC migration (Li Q. et al., 2017). The limitations of the Lit team’s research lie in the use of an IL-6-stimulated *in vitro* model, which differs from the pathological processes induced by hypoxia. In contrast, the therapeutic effect of metabolite magnolol from Magnolia officinalis Rehder and E.H.Wilson on pulmonary hypertension was contrary to that of DHM. Magnolol can reduce the expression of myocardial fibrosis markers such as collagen I, III, and α-SMA, reverse the increase in elevated p-JAK2 and p-STAT3 in hypoxic rat right ventricular tissue, and also ameliorate the cardiac fibroblast fibrosis and right ventricular hypertrophy in PAH rats (Chen L. et al., 2021; Chen M. et al., 2021). In this study, magnolol was shown to attenuate right ventricular remodeling. Moreover, the use of JAK2 inhibitors supported involvement of the JAK2/STAT3 pathway, thereby strengthening the mechanistic evidence. However, the *in vitro* experiments employed H9c2 rat cardiomyoblasts rather than primary cardiomyocytes. These cells differ from mature cardiomyocytes in differentiation status and functional properties, which may limit physiological relevance.

Ginsenoside Rg1, a major purified active metabolite from ginseng, ameliorates PAH. Treatment with Rg1 improved the viability of PASMCs and downregulated the expression levels of major proteins, including calpain-1, p-STAT3, IL-6, TGF-β1, and PCNA. Calpain-1 is the major member of the calpain family and is a calcium-activated cysteine protease (Zhang et al., 2021). It was reported that calpeptin attenuates the development of HPH by decreasing the mRNA levels of IL-6, Ang-1, TGF-β1, and COL1A1 (collagen synthesis gene) in lung tissues of bleomycin-treated mice (Tabata et al., 2010). Ultimately, Rg1 suppresses the inflammatory response, cell proliferation, and fibrotic process to attenuate the pulmonary vascular remodeling induced by hypoxia (Ran et al., 2024). This study features a rigorous design, employing both calpain-1 knockout mice and pharmacologic calpain-1 inhibition to validate the mechanism. Furthermore, calpain-1 overexpression experiments provided bidirectional evidence, complementing loss-of-function (deletion/inhibition) with gain-of-function approaches, thereby supporting a causal role for calpain-1 in hypoxia-induced PASMC proliferation. Consequently, compared with studies relying solely on inhibitors or lacking genetic suppression, this work provides stronger causal inference regarding Rg1’s mechanism of action.

### 4.6 NLRP3 signaling pathway

NLRP inflammasomes are intracellular heteromeric oligomeric complexes that participate in innate immunity. NLRP genes are predominantly expressed in the cytoplasm of macrophages and provide a rapid and efficient response to DAMP released by infected, stressed, dying, and senescent cells, as well as PAMP from bacterial and viral infections. By recognizing signals of infection or tissue injury (PAMPs and DAMPs), the activated inflammasomes promote the production of proinflammatory cytokines, which recruit immune cells to the site of infection and injury, induce inflammation, and promote tissue and organ repair (Xu et al., 2024) (Table 6). The NLRP3 inflammasome is a cytoplasmic immune factor responding to cellular stress signals. When the NLRP3 inflammasome is activated by danger signals released from the host or pathogens, proinflammatory cytokines IL-1β and IL-18 are secreted, and inflammatory cell pyroptosis is induced (Guan et al., 2022). Expression of NLRP3 inflammasome was activated in a mouse model of hypoxic PAH, and targeting this inflammasome is an effective approach for PH (Villegas et al., 2013).

**TABLE 6:** Natural products acting via the NLRP3 signaling pathway.

Natural products acting via the NLRP3 signalling pathway								
Natural products	Model used	Species	Model’s induction method	Intervention doses	Administration	Periods	Efficacy	References
Astragaloside IV	*In vivo*	Rat	MCT (60 mg/kg,i.p.)-induced PAH rats	AS-IV 40/80 mg/kg	*i.g*	28 days	Inhibiting the inflammatory response	Sun et al. (2021)
	*In vitro*	HPAECs	MCTP (60 μg/mL 24 h)-induced HPAECs	AS-IV 50/100 μmol/L	*—*	30 min		
	*In vivo*	Mice	Hypoxia-induced PH mice	—	*—*	—	Reversing hypoxia-induced elevation of pulmonary artery pressure and impairment of endothelium-dependent relaxation	Niu et al. (2025)
	*In vitro*	HPAECs	Hypoxia-induced HPAECs	—	*—*	—		
Ellagic acid	*In vivo*	Rat	MCT-induced PAH rats	EA 30/50 mg/kg/day	*i.g*	4 weeks	Reduce RVSP, RVH, and the ratio of pulmonary artery wall thickness to outer diameter	Tang et al. (2015)
Safflower	*In vivo*	Rat	MCT (60 mg/kg,s.c.)-induced PAH rats	SI 2 mL/kg/day	*i.p*	20 days	Inhibiting right ventricular hypertrophy whilst improving pulmonary vascular remodeling	Ding et al. (2024)
	*In vitro*	PASMCs	MCT (1 μmol/mL,24 h)-induced PAH PASMCs and PAECs	SI 200 μg/mL	*—*	48 h		
Rutin	*In vivo*	Rat	MCT (60 mg/kg,i.p.)-induced PAH rats	Rutin 200 mg/kg/day	*i.g*	21 days	Improving MCT-induced RVSP and RV/LV + S	Che et al. (2024)
	*In vitro*	PASMCs	Hypoxia-induced PASMCs	Rutin 0.1 μmol/L	*—*	—		

The botanical drug Carthamus tinctorius L. (safflower in Traditional Chinese Medicine) exhibits multiple pharmacological effects, such as anti-inflammatory and antioxidant effects (Alshareef et al., 2024; Dong et al., 2024). It can modulate pulmonary arterial remodeling, which may affect pulmonary arteriolar wall thickness and myocardial hypertrophy, and then ameliorate the pathological condition of PH. Network pharmacology and molecular docking results indicated that quercetin is the main metabolite in safflower, which could delay the progression of PAH by inhibiting the NLRP3 inflammasomes. The study group validated the network pharmacology results of safflower using *in vivo* and *in vitro* experiments (Ding et al., 2024). This study employed a multidimensional research strategy integrating network pharmacology, molecular docking, and experimental validation. Screening identified 15 active metabolites and 177 putative targets, with KEGG pathway enrichment analysis indicating multiple pathways—including the TNF signaling pathway and Th17 cell differentiation—underpinning safflower’s anti-inflammatory effects. Subsequent animal experiments confirmed that safflower exerts its anti-inflammatory effects by inhibiting NLRP3 inflammasome activation.

Furthermore, in the present study, we found that the natural triterpene AS-IV and the Ellagic acid (EA) exhibited the same effect as safflower. AS-IV and EA could suppress IL-8 and IL-1β expression by inhibiting NLRP3 inflammasome activation, which ameliorated MCT-induced PAH, pulmonary vascular remodeling, and right ventricular hypertrophy (Sun et al., 2021; Tang et al., 2015). Tang and colleagues were among the first to investigate the role of the NLRP3 inflammasome in PH pathogenesis, providing foundational evidence. Building on this work, Sun and colleagues further elucidated how calpain-1 functions as an upstream regulator of the NLRP3 inflammasome. The study employed an integrated *in vivo* and *in vitro* design, strengthening the robustness of its conclusions. Furthermore, pharmacologic inhibition of NLRP3 and calpain-1 provided convergent evidence that AS-IV exerts anti-inflammatory effects by inhibiting the calpain-1–NLRP3 axis.

Increasing evidence revealed that ferroptosis and NLRP3 inflammasome exhibit remarkable crosstalk in the pathogenesis of PAH, and their mutual activation can synergistically promote the development of PAH (Xie et al., 2022). Mechanistically, NLRP3 attenuates the expression of the vital antioxidant enzyme GPX4 and then promotes the lipid peroxidation–induced ferroptotic cell death by enhancing the lipid peroxidation process and potentiating pulmonary vascular remodeling (He et al., 2024; Wang J. et al., 2024). Whereas, ferroptotic cell death releases high-mobility group box 1 (HMGB1) to recruit and activate the NLRP3 inflammasome and then enhances local inflammatory cascade, increases pulmonary arterial pressure and right ventricular afterload—forming a positive feedback loop (Jiang et al., 2025). Accordingly, inhibition of PAH progression by suppressing ferroptosis has been proposed as a novel therapeutic strategy. In addition, rutin, as a widely distributed plant-derived dietary flavonoid, could attenuate PAH models via regulating the conformation and catalytic activity of protein kinase Cα (PKCα) and then suppresses ferroptotic response (Che et al., 2024). In contrast to rutin, astragaloside IV attenuates hypoxia-induced pulmonary endothelial dysfunction via inhibiting the calpain-1/TGF-β/TFRC signaling axis and then attenuates iron overload and ferroptosis-induced injury (Niu et al., 2025).

### 4.7 Other signaling pathway

In addition to the inflammation-related pathways described above, other signaling pathways regulated by natural products may also exert therapeutic effects PH. Oxidative stress and the accumulation of reactive oxygen species (ROS) play a significant role in the pathogenesis of PH. Profusely generated ROS promotes immune cell activation and inflammatory cytokine production, thereby activating pro-inflammatory signaling pathways and establishing a feed-forward vicious cycle within the vasculature, which in turn drives pulmonary vascular remodeling and right ventricular hypertrophy (Aboukhater et al., 2023; Xu et al., 2022). Nuclear factor erythroid 2–related factor 2 (Nrf2) is a key transcription factor that maintains intracellular redox homeostasis and represents a crucial defence mechanism against oxidative stress. Under normal conditions, Nrf2 is sequestered in the cytoplasm by Kelch-like ECH-associated protein 1 (Keap1) and targeted for degradation via the ubiquitin–proteasome pathway, thereby maintaining low Nrf2 levels (Wang et al., 2023). Research has demonstrated that various natural products can activate Nrf2, dissociate it from its inhibitory partner Keap1, promote its translocation to the nucleus, and facilitate its binding to antioxidant response element (ARE) sequences. This in turn induces the expression of a series of antioxidant enzymes, thereby reducing ROS levels, alleviating oxidative stress, and attenuating PAH progression. Puerarin acid (PA) has been found to inhibit cellular ROS accumulation by activating the Nrf2–Keap1–ARE signaling pathway in hypoxic PASMCs, thereby ameliorating PH in experimental models (He Y. et al., 2022). Salidroside (SAL), an active metabolite of Rhodiola rosea L, primarily exerts its effects by promoting Nrf2 nuclear translocation and enhancing the expression of downstream antioxidant proteins haem oxygenase-1 (HO-1) and NAD(P)H quinone oxidoreductase 1 (NQO1) in pulmonary arterial endothelial cells (PAECs). This alleviates hypoxia-induced oxidative stress in PAECs, reduces right ventricular systolic pressure in hypoxia-exposed rats, and attenuates pulmonary vascular remodeling and right ventricular hypertrophy (Lei et al., 2024). In contrast to salidroside, andrographolide acts through dual mechanisms, both inhibiting NADPH oxidase (NOX) activation and enhancing Nrf2 expression. Both *in vitro* and *in vivo* studies have confirmed that andrographolide suppresses hypoxia-triggered ROS production, modulates Nrf2-mediated oxidative stress responses, and attenuates pulmonary vascular remodeling (Nie et al., 2021). Regarding pathological outcomes, all three metabolites demonstrated efficacy in a hypoxia-induced PH animal model, as evidenced by attenuated pulmonary vascular remodeling, reduced right ventricular hypertrophy, and improved hemodynamic parameters. Consistent with the limitations noted above, these studies lacked clinical trial data, and key pharmacokinetic parameters—including bioavailability and plasma half-life—were not fully characterized. Consequently, the optimal therapeutic dose, route of administration, and treatment duration cannot be defined (Table 7).

**TABLE 7:** Natural products acting via the Nrf2 signaling pathway.

Natural products acting via the Nrf2 signalling pathway								
Natural products	Model used	Species	Model’s induction method	Intervention doses	Administration	Periods	Efficacy	References
Andrographolide	*In vivo*	*C57BL/6J*	Mice were subjected to either chronic hypoxia alone or a combined chronic hypoxia with i.p. injection of semaxanib	Andrographolide 1 mg/kg/day	*i.p*	4 weeks	Reducing distal PA remodeling, mPAP, and RVH.It also diminished the viability, proliferation, and migration of PASMCs isolated from PH patients, whilst increasing cellular apoptosis	Nie et al. (2021)
	*In vitro*	HPASMCs	Human PASMCs were isolated from the lung tissues of normal donors and patients with PH	ANDRO 1/3/10/30/60/100 μM	*—*	24 h		
Salidroside	*In vivo*	Rat	Hypoxia-induced PH rats	SAL 2/8/32 mg/kg/d	*i.p*	4 weeks	This improvement was accompanied by a reduction in right ventricular pressure, pulmonary vascular remodeling, and right ventricular hypertrophy	Lei et al. (2024)
Pachymic acid	*In vivo*	Rat	Hypoxia-induced PH rats	PA 5mg/lg	*—*	4 weeks	Alleviating PH and hypoxia induced pulmonary vascular Remodeling. Alleviating hypoxia-induced Oxidative stress in PASMCs	He Y. et al. (2022)
	*In vitro*	PASMCs	Hypoxia-induced PASMCs	PA 2.5/5/10 μmol/L	*—*	24 h		

In pulmonary arterial hypertension (PAH), excessive proliferation of pulmonary arterial smooth muscle cells (PASMCs) and vascular remodeling constitute core pathological features, in which multiple cytokines—particularly transforming growth factor-β1 (TGF-β1)—play pivotal roles (Liu et al., 2024). Experimental studies indicate that TGF-β1 is significantly upregulated in PAH models. Upon binding to its receptors (TβRI/TβRII), TGF-β1 aberrantly activates the downstream Smad2/3 pathway while concurrently suppressing Smad1/5/8 signaling. This drives PASMC proliferation, migration, and extracellular matrix deposition, thereby leading to vascular wall thickening and luminal narrowing (Dhananjayan et al., 2025). The TGF-β1/Smad pathway thus represents a pivotal signaling axis driving inflammation, fibrosis, and vascular remodeling. Natural products, as potential modulators of the TGF-β pathway, are hypothesised to offer novel therapeutic strategies for alleviating PAH by restoring the balance between Smad2/3 and Smad1/5/8, thereby inhibiting abnormal proliferation and vascular remodeling. Alginate oligosaccharides (AOS) have been shown to inhibit monocrotaline (MCT)-induced pulmonary vascular remodeling by suppressing the TGF-β1/p-Smad2 signaling pathway. Furthermore, studies have revealed that AOS concurrently downregulate pro-inflammatory cytokine expression, reduce macrophage infiltration, and upregulate anti-inflammatory cytokine expression. These actions collectively exert antioxidant and anti-inflammatory effects, thereby mitigating the progression of PAH (Feng et al., 2020). Moreover, osthole and Danshensu, which share a mechanism similar to that of AOS, inhibit abnormal proliferation of pulmonary arterial smooth muscle cells (PASMCs) by suppressing the TGF-β1/Smad signaling pathway, thereby emerging as promising natural therapeutic candidates for PAH (Yue et al., 2020; Zhang N. et al., 2018). However, Yue and colleagues performed only *in vitro* experiments, which suggest that osthole inhibits PASMC proliferation by modulating the TGF-β1/Smad/p38 signaling pathway. PH pathogenesis involves complex interactions among multiple cell types, and single-cell culture systems cannot recapitulate multicellular paracrine cross-talk. Accordingly, these findings should be interpreted as preliminary mechanistic evidence. To further evaluate therapeutic potential and translational relevance, *in vivo* animal studies are required. By contrast, Feng and colleagues’ therapeutic dosing regimen may offer greater clinical translatability (Table 8).

**TABLE 8:** Natural products acting via the TGF-β/Smad signaling pathway.

Natural products acting via the TGF-β/Smad signalling pathway								
Natural products	Model used	Species	Model’s induction method	Intervention doses	Administration	Periods	Efficacy	References
Alginate oligosaccharide	*In vivo*	Rat	MCT (60 mg/kg,i.p.)-induced PAH rats	AOS 5/10/20 mg/kg/day	*i.p*	3 weeks	AOS prevents MCT-induced pulmonary vascular remodeling	Feng et al. (2020)
Danshensu	*In vivo*	Rat	Hypoxia-induced PH rats	DSS 160 mg/kg/day	*i.p*	4 weeks	Danshensu significantly decreased the RVSP, the RVH, and the pulmonary vascular remodeling index in hypoxic pulmonary hypertension rats	Zhang N. et al. (2018)
	*In vitro*	PASMCs		—	*—*	—		

The RhoA/Rho-associated coiled-coil-containing protein kinase (ROCK) pathway is recognised as a key signaling axis in PAH pathogenesis and progression. Activation of RhoA leads to downstream ROCK activation, promoting pulmonary arterial smooth muscle cell (PASMC) contraction, proliferation, and vascular remodeling, thereby exacerbating pulmonary hypertension/PAH (Yeganeh et al., 2014). The bioactive alkaloid aloperine and 18β-glycyrrhetinic acid have been found to inhibit monocrotaline (MCT)-induced pulmonary hypertension in rats by modulating the RhoA/ROCK pathway. Furthermore, *in vitro* studies have demonstrated that 18β-glycyrrhetinic acid significantly suppresses PDGF-BB-induced proliferation of human pulmonary arterial smooth muscle cells (HPASMCs) (Wu et al., 2017; Zhang M. et al., 2019). As an active metabolite of licorice, 18β-GA is widely used in humans and readily accessible. However, licorice metabolites may induce pressor effects, sodium and water retention, and electrolyte disturbances, resembling mineralocorticoid-like adverse reactions, which are potentially problematic in PAH, given the predisposition to right-sided heart failure. Consequently, it is essential to define the dose–exposure–risk relationship during translational development (Table 9).

**TABLE 9:** Natural products acting via the RhoA/Rho signaling pathway.

Natural products acting via the RhoA/Rho signalling pathway								
Natural products	Model used	Species	Model’s induction method	Intervention doses	Administration	Periods	Efficacy	References
18β-glycyrrhetinic acid	*In vivo*	Rat	MCT (60 mg/kg,i.p.)-induced PAH rats	18β-GA 100/50/25 mg/kg/day	*i.g*	—	Reducing mPAP, RVSP, RVHI and inhibiting pulmonary arteriolar remodeling	Zhang T. et al. (2019)
	*In vitro*	HPASMCs	PDGF-BB(20 ng/mL)-induced HPASMCs	18β-GA 20/40/80/160 μM	*—*	24 h		
Aloperin	*In vivo*	Rat	MCT (60 mg/kg,s.c.)-induced PAH rats	Aloperin 100/50/25 mg/kg/day	*i.g*	21 days	Reducing the pulmonary artery pressure, right ventricular pressure, and right ventricular hypertrophy in rats with pulmonary hypertension	Wu et al. (2017)

## 5 Application prospects and strategies for natural products

### 5.1 Pharmacokinetics of natural products

The metabolic processes of natural products within the body represent a critical determinant of their therapeutic efficacy. While most natural products are administered orally, their bioavailability is generally low due to rapid metabolism in the intestines and liver, thereby limiting their *in vivo* therapeutic efficacy. For instance, studies indicate that following oral administration of green tea polyphenols, only a small fraction is absorbed in the small intestine and subsequently taken up by the liver. Subsequently, a portion enters the enterohepatic circulation, from which it may be reabsorbed or ultimately excreted in the urine (Cai et al., 2018). Resveratrol, another polyphenolic metabolite akin to green tea polyphenols, also exhibits low oral bioavailability. Although studies indicate approximately 75% oral absorption, the majority undergoes extensive first-pass metabolism in the gut and liver and persists in the body predominantly as glucuronide and sulphate conjugates. This results in low circulating concentrations of free resveratrol in plasma (Springer and Moco, 2019). The natural flavonoid quercetin faces similar challenges in therapeutic applications, primarily existing in the body as conjugates such as quercetin glucuronide and quercetin sulphate, which hinders the realisation of its full therapeutic potential (Lotito et al., 2011). Thus, despite the significant biological activity of such natural products, their clinical translation is substantially constrained by low oral bioavailability. To address this issue, an increasing body of research is focused on enhancing *in vivo* exposure and bioavailability through strategies such as formulation optimisation and alternative delivery routes.

### 5.2 Safety of natural products

Many natural products have been used for centuries across medical and ethnomedicinal traditions, providing a substantial empirical foundation. In recent years, animal studies and limited clinical observational evidence have suggested that some natural products may have therapeutic efficacy and acceptable safety profiles. However, the evidence base remains dominated by preclinical studies, and both the volume and methodological quality of clinical evidence require improvement. Well-designed clinical studies are urgently needed, and systematic reviews and meta-analyses should be used to appraise study quality and synthesize findings. Together, these efforts will more reliably establish the efficacy and safety of natural products for pulmonary arterial hypertension (PAH). In parallel, to support broader clinical application, a robust safety-assessment and regulatory framework should be established, incorporating tiered evaluations by relevant authorities—laboratory testing, animal toxicology, and clinical trials—to assess potential risks to human health.

### 5.3 Improved strategies for enhancing the utilisation of natural products

#### 5.3.1 Nanomaterial delivery systems

Nanomaterial systems, as an advanced drug delivery platform, have demonstrated significant potential in the delivery and therapeutic application of natural products. These systems encompass liposomes, solid lipid nanoparticles (SLNs), nanoemulsions/nanomulsions, nanomicelles, polymeric nanoparticles, and other related nanocarriers. By encapsulating natural bioactive metabolites such as polyphenols, flavonoids, and resveratrol, nanomaterial-based delivery systems markedly enhance the solubility and stability of these metabolites in aqueous media and bodily fluids (Pathak et al., 2025). Nanocarriers effectively shield active metabolites from degradation or rapid metabolism within the gastrointestinal tract while prolonging their circulation time *in vivo*, thereby enhancing bioavailability via oral or alternative administration routes. Crucially, nanostructures can be engineered to enhance cellular and tissue permeability by adjusting their size and surface properties, thereby ensuring targeted drug release at specific sites and minimising adverse effects (Chakraborty et al., 2023).

Clinically, nanomaterial systems address numerous challenges that are difficult to overcome with conventional drug delivery methods, particularly for natural products exhibiting poor solubility, instability, and low bioavailability. For instance, employing polymeric microparticles or other polymeric carriers enables controlled drug release, ensuring precise delivery to target areas while minimising systemic drug fluctuations and adverse reactions. As an example, nano-spray drying technology has been employed to deliver drugs to the lungs, enhancing pulmonary deposition through controlled-release formulations based on polymeric microparticles (Naz et al., 2022). Such innovative techniques enable more precise delivery of natural products for the treatment of conditions such as pulmonary arterial hypertension.

#### 5.3.2 Structural modification

To enhance the bioavailability of natural products, researchers have adopted a range of innovative strategies in recent years. These approaches include chemical modification and prodrug design, which aim to overcome the inherently low bioavailability of many natural products by altering their structures to improve stability, absorption, and biological activity *in vivo* (Ahmed et al., 2025). Chemical modification techniques improve lipophilicity, stability, and membrane permeability through covalent conjugation of functional groups such as lipids, glycosides, amino acids, or peptides. These modifications can reduce metabolic rates and prolong the duration of action *in vivo* (Xu et al., 2021). Furthermore, structural optimisation and modulation of solubility can significantly enhance the bioavailability and biological activity of natural products, thereby helping to address the suboptimal therapeutic efficacy of many traditional natural products arising from low solubility and rapid metabolism.

These strategies have been widely applied, particularly to flavonoid metabolites. Research on flavonoid derivatives has revealed that hydrophobic benzyl substitution on the B-ring, coupled with modification of the 7-OH group, can confer superior inhibitory activity compared to the parent flavonoid (Yang et al., 2016). Furthermore, it has been demonstrated that converting the hydroxyl groups of quercetin, apigenin, and flavonol glycosides into acetamido groups increases their bioavailability relative to metabolites derived from the parent plant (Hu et al., 2025). Moreover, structural modification to enhance bioavailability is increasingly being exploited in the development of novel therapeutics based on natural products. Notably, a modified form of chrysin, developed for adult influenza treatment, has progressed to Phase II clinical trials (Zhao et al., 2022). Through chemical modification and prodrug design, the pharmacokinetic properties of natural products can be optimised, thereby potentially improving their therapeutic efficacy. These advances provide robust support for the further clinical development of natural products and offer novel insights into their more effective utilisation in the future.

## 6 Discussion

In the above sections, the present situation of pulmonary hypertension (PH) morphology, pathogenesis, and mechanism of effective natural product-based therapies has been briefly reviewed. Then, this study initially reveals the basic definition of PH. Pulmonary hypertension is a pulmonary vascular disease characterized by structural remodeling of distal pulmonary arterioles. Pathological phenotypes such as intimal hyperplasia, hypertrophy of the smooth muscle layer, activation of adventitial fibroblasts, excessive extracellular matrix deposition, etc., gradually lead to the narrowing of the lumen and occlusion of small pulmonary arteries. All of these changes are closely related to the proliferation and apoptosis resistance of endothelial and smooth muscle cells (Humbert et al., 2019).

In addition to the above pathological phenotypes, the process of disease development is accompanied by a persistent increase in pulmonary vascular resistance, abnormally increased pressure in pulmonary arterial circulation, vascular stenosis, and occlusion of the pulmonary vascular bed. In order to counteract the above changes, which lead to increased afterload, the right ventricle will adaptively hypertrophy initially. However, chronic overload will finally induce right ventricular failure, which is the main cause of mortality in patients with PH (Thenappan et al., 2018). Currently, available pharmacological therapies for PH can only alleviate the clinical symptoms of patients partially, but are still unable to reverse pulmonary vascular remodeling effectively. Therefore, it is an urgent task to develop novel, highly effective, and safe therapeutic agents for PH.

Secondly, this study will further focus on the important role of inflammation in the pathological process of pulmonary hypertension (PH). When pulmonary vascular endothelial cells are stimulated by pathological stimuli or endothelial injury occurs, the recruitment and adhesion of inflammatory cells to the vascular wall will be promoted. The inflammatory cells infiltrating into the vessel will finally release various cytokines and inflammatory factors, and participate in the process of pathogenesis of PH through the following mechanisms. On the one hand, they will damage the endothelial barrier and affect the physiological function of the endothelial cells. On the other hand, they will promote the migration and hyperproliferation of smooth muscle cells and induce the collagen fiber deposition in the fibrotic process (Huertas et al., 2020).

The synergistic actions of the above pathological phenomena induce the pulmonary vascular remodeling and thereby promote the occurrence and development of pulmonary hypertension. Therefore, eliminating vascular inflammation by targeting inflammatory pathways has become a new strategy for the treatment of pulmonary hypertension.

Thirdly, it has been clinically observed and experimentally confirmed that the therapeutic effect of natural products on pulmonary hypertension has gradually been recognized in recent years. As a rich source of multi-targeted activity and low toxicity, natural products have obtained new therapeutic advantages in treating pulmonary hypertension. Owing to the above characteristics, this study initially believes that natural products have the potential to be developed as novel therapeutic agents for pulmonary hypertension.

A number of bioactive compounds extracted from natural products have been reported to exhibit protective effects against the development and progression of PH, such as tea polyphenols, resveratrol, naringenin, tanshinone, baicalin, baicalein, astragaloside IV, astragalus polysaccharides, proanthocyanidins from grape seeds, silybin, hesperidin, oxymatrine, α-solanine, ellagic acid, dihydromyricetin, capsaicin, cinnamaldehyde, and andrographolide, among others. The chemical structure of the natural product used for treating PH is shown in Table 10.

**TABLE 10:** Sources and molecular Formulas of natural products.

Natural products	Source	Chemical structure	Pathways	References
Baicalin	*Scutellaria baicalensis Georgi*	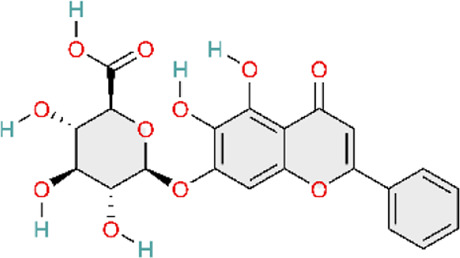	NFκB, PI3K	Luan et al. (2015); Yan et al. (2019); Xue et al. (2021); Huang et al. (2017); Zhang et al. (2014)
Baicalein	*Scutellaria baicalensis Georgi*	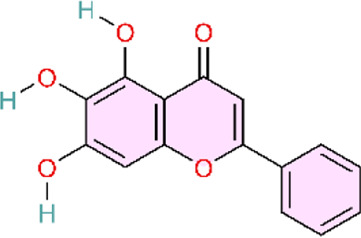	NFκB	Shi et al. (2018a); Shi et al. (2018b)
Grape seed proanthocyanidin	*Vitis vinifera L*	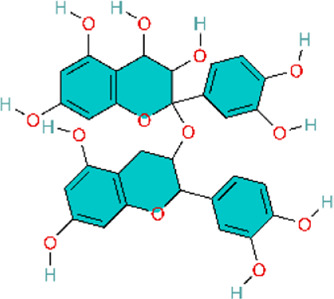	NFκB, PPARγ	Chen et al. (2018); Jin et al. (2016); Liu J. et al. (2020)
Cinnamaldehyde	*Cinnamomum verum J.Presl*	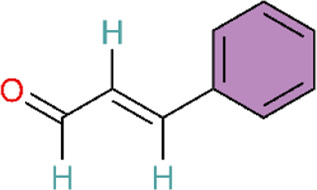	NFκB	Zhang et al. (2025)
Gypenoside	*Gynostemma pentaphyllum (Thunb.) Makino*	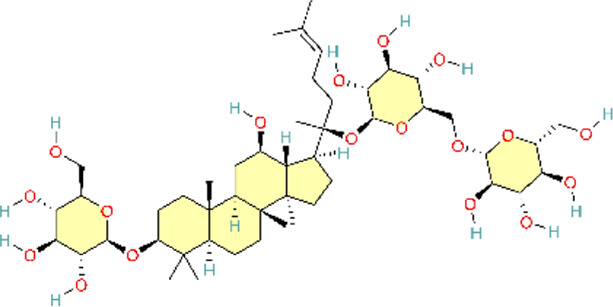	NFκB	Du et al. (2025)
18β-glycyrrhetinic acid	*Glycyrrhiza glabra L*	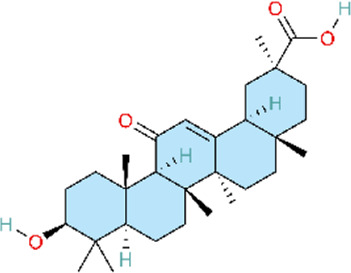	NFκB, RhoA/Rho	Zhang T. et al. (2019); Wang et al. (2022)
Hesperidin	*Citrus × aurantium L*	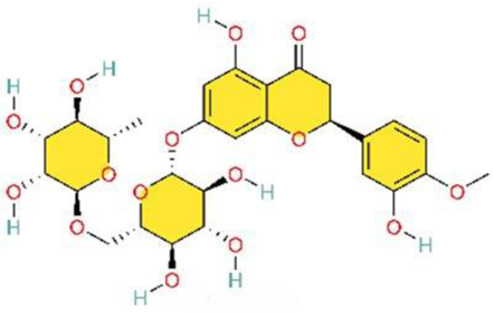	NFκB	Fang et al. (2025); He and Liao (2025)
Andrographolide	*Andrographis paniculata (Burm.f.) Wall. ex Nees*	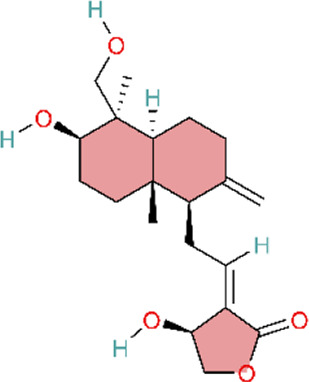	NFκB, Nrf2	Nie et al. (2021)
Osthole	*Cnidium monnieri (L.) Cusson*	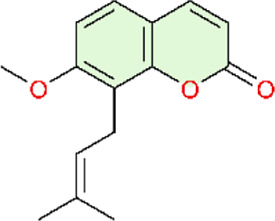	NFκB	Li Y. et al. (2017)
Curcumin	*Curcuma longa L*	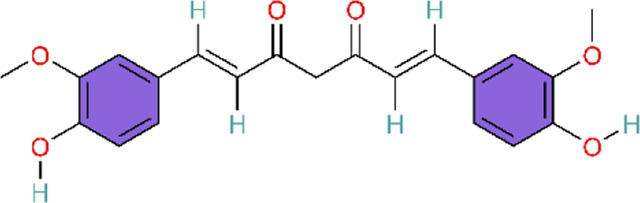	NFκB	Rice et al. (2016)
Betaine	*Beta vulgaris L*	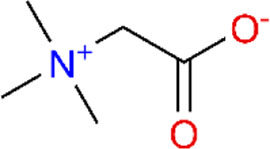	NFκB	Yang et al. (2018)
Resveratrol	*Reynoutria japonica Houtt*	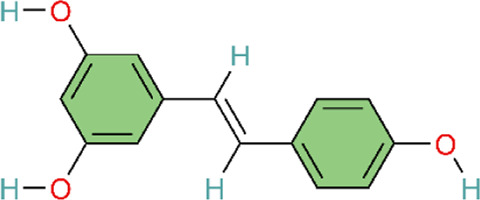	NFκB, PI3K/AKT, MAPK	Shi et al., 2018c; Xu et al. (2016); Chen et al. (2014); Guan et al. (2017); Chun et al. (2012)
Astragalus polysaccharides	*Astragalus mongholicus Bunge*	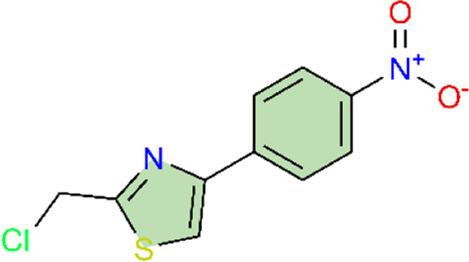	NFκB	Yuan et al. (2017)
Nobiletin	*Citrus reticulata Blanco*	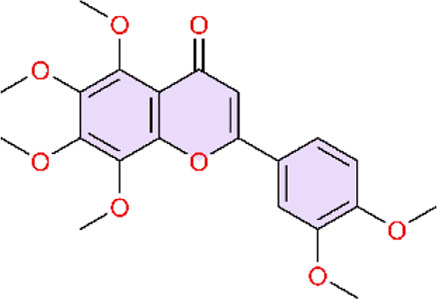	PI3K/AKT	Yin et al. (2023)
Sodium tanshinone IIA sulfonate	*Salvia miltiorrhiza Bunge*	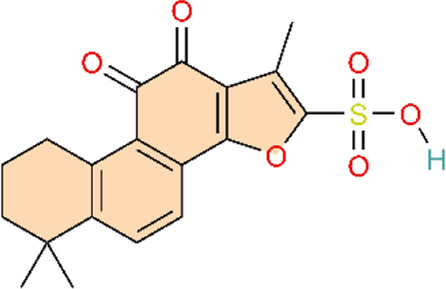	PI3K/AKT, PPAR-γ	Bao et al. (2020); Jiang et al. (2016)
Ligustrazine	*Conioselinum anthriscoides ‘Chuanxiong'*	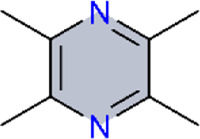	PI3K/AKT	Huang et al. (2021)
α-Solanine	*Solanum tuberosum L*	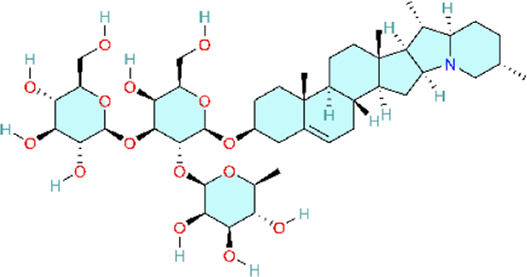	PI3K/AKT	Nie et al. (2017)
Isoquercitrin	*Betula pendula subsp. mandshurica (Regel) Ashburner and McAll*	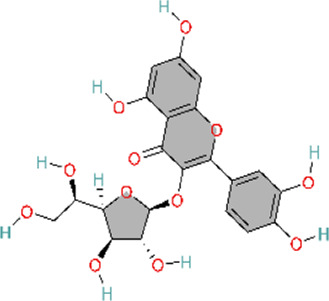	PI3K/AKT	Zhang et al. (2017)
Genistein	*Glycine max (L.) Merr*	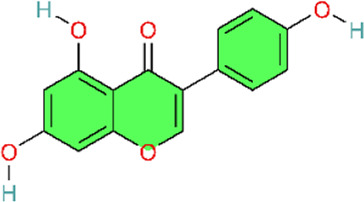	PI3K/AKT	Kuriyama et al. (2014)
Astragaloside IV	*Astragalus mongholicus Bunge*	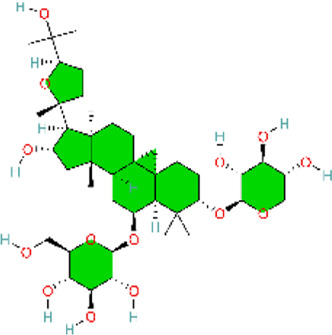	PI3K/AKT, MAPK, NLRP3	XinTian et al. (2022); Sun et al. (2021); Niu et al. (2025); Jin et al. (2021)
Dihydroartemisinin	*Artemisia annua L*	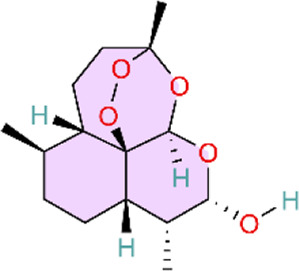	PI3K/AKT	Tang et al. (2020)
Aloperine	*Sophora alopecuroides L*	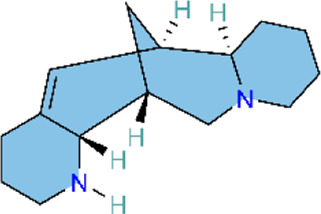	PPARγ	Shan et al. (2023)
Puerarin-V	*Pueraria montana* var. *lobata (Willd.) Maesen and S.M.Almeida ex Sanjappa & Predeep*	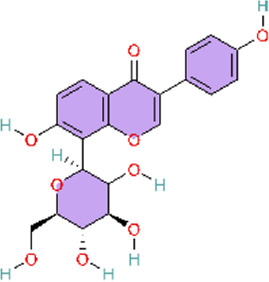	PPARγ	Chen et al. (2022)
Magnesium lithospermate B	*Salvia miltiorrhiza Bunge*	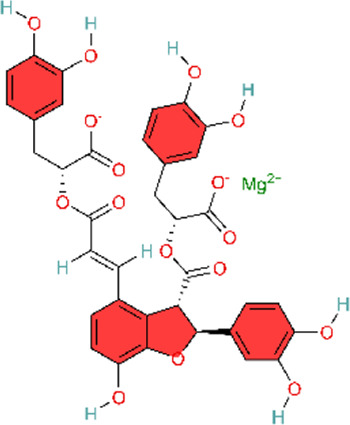	MAPK	Qu et al. (2020)
Capsaicin	*Capsicum annuum L*	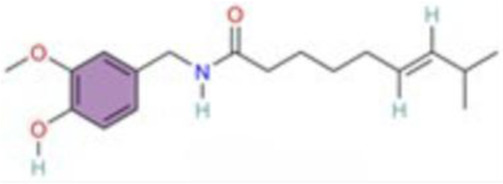	MAPK	Xu et al. (2017)
Ginsenoside	*Panax ginseng C.A.Mey*	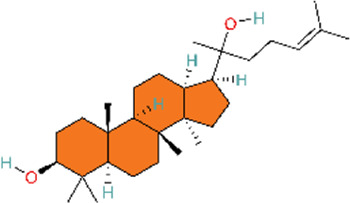	MAPK	Qin et al. (2016); Ran et al. (2024)
Paeonol	*Paeonia lactiflora Pall*	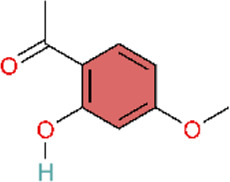	MAPK	Zhang L. et al. (2018)
Magnolol	*Magnolia officinalis Rehder and E.H.Wilson*	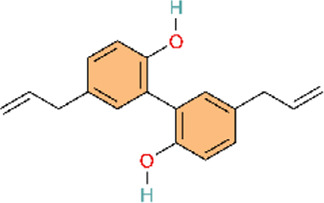	JAK, STAT	Fu M. et al. (2021)
Dihydromyricetin	*Hovenia dulcis Thunb*	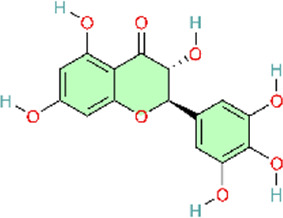	JAK, STAT	Li Q. et al. (2017)
Ellagic acid	*Punica granatum L*	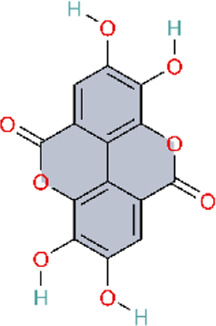	NLRP3	Tang et al. (2015)
Safflower	*Carthamus tinctorius L*	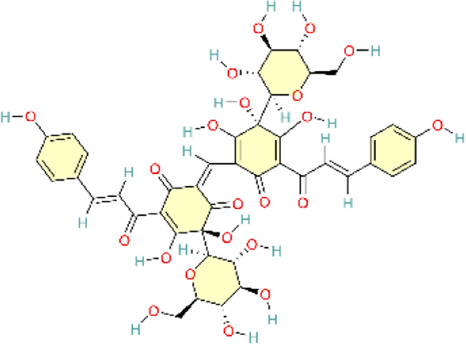	NLRP3	Ding et al. (2024)
Rutin	*Fagopyrum esculentum Moench*	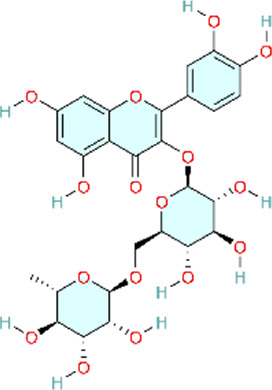	NLRP3	Che et al. (2024)
Salidroside	*Rhodiola rosea L*	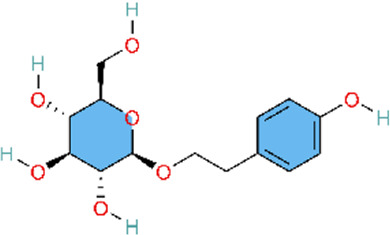	Nrf2	Lei et al. (2024)
Pachymic acid	*Wolfiporia extensa (Peck) Ginns*	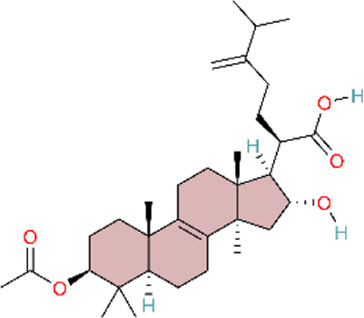	Nrf2	He Y. et al. (2022)
Danshensu	*Salvia miltiorrhiza Bunge*	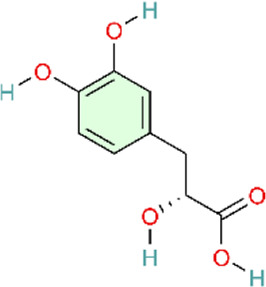	TGF-β/Smad	Zhang N. et al. (2018)
Naringenin	*Citrus × limon (L.) Osbeck*	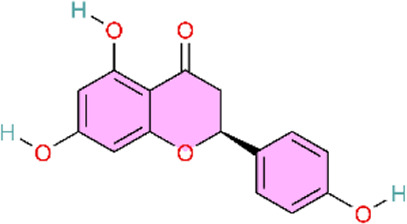	NO/eNOS	Ahmed et al. (2014)
Kaempferol	*Camellia sinensis (L.) Kuntze*	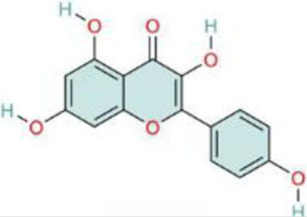	—	Yi et al. (2022)

Among numerous natural products, the therapeutic effects of baicalein and baicalin extracted from Scutellaria baicalensis Georgi are particularly notable. Scutellaria baicalensis Georgi has been shown to exhibit broad anti-inflammatory and antioxidant properties (Liao et al., 2021). Moreover, extracts of S. baicalensis can significantly reduce inflammatory responses by modulating key signaling pathways such as NF-κB and MAPK, thereby demonstrating substantial potential in the prevention and treatment of pulmonary hypertension (PH). Regarding safety, clinical multiple-dose escalation trials have indicated that repeated oral administration of baicalin within the 200–800 mg range is associated with favourable pharmacokinetic characteristics and good tolerability, with no apparent renal or hepatic toxicity observed. This provides important evidence for its further development (Li et al., 2014). However, because of baicalin’s inherently low oral bioavailability, researchers have explored the development of nano- and microscale baicalin delivery systems to enhance its *in vivo* absorption and therapeutic efficacy (Huang et al., 2019). Considering the aforementioned pharmacological basis, mechanistic studies, and clinical safety evidence, baicalin, baicalein, and their derivatives—particularly investigations focusing on optimising mechanisms of action and delivery systems targeting inflammation- and stress-related signaling pathways such as NF-κB and MAPK—may be regarded as priority directions for in-depth advancement in the field of PH intervention.

Beyond the flavonoid metabolites associated with Scutellaria baicalensis Georgi, the polyphenolic metabolite resveratrol has also demonstrated significant therapeutic potential in the prevention and treatment of pulmonary hypertension (PH). On the one hand, resveratrol mitigates hypoxia-induced oxidative stress and the proliferation and migration of pulmonary vascular cells. On the other hand, it significantly inhibits pulmonary vascular inflammation and remodeling by suppressing SphK1/S1P-mediated NF-κB activation and the expression of downstream inflammatory mediators such as monocyte chemoattractant protein-1 (MCP-1) (Liu Y. Y. et al., 2020; Shi et al., 2018c). Traditional PH therapies primarily employ intravenous and oral routes, which can readily lead to systemic adverse reactions, including hepatic injury, pain, diarrhoea, nausea, and dyspnoea. Inhalation delivery, being non-invasive, painless, and capable of enhancing local drug concentrations and bioavailability, is regarded as a promising alternative. To further enhance therapeutic efficacy and achieve controlled release, researchers have explored carrier systems such as polymeric microparticles to formulate resveratrol as a dry powder suitable for pulmonary delivery. Among these, technology developed by Buchi Labortechnik enables the preparation of dry powders with aerodynamic diameters below 5 μm, facilitating efficient deposition in the lower respiratory tract. Nano-spray dryers, in particular, yield powders with smaller particle sizes and higher yields, thereby potentially enhancing resveratrol’s overall therapeutic efficacy (van der Koog et al., 2022). Taken together with its characteristic multi-target profile, which enables synergistic actions through multiple signaling pathways, resveratrol holds substantial translational potential in the treatment of PH.

**FIGURE 5:**
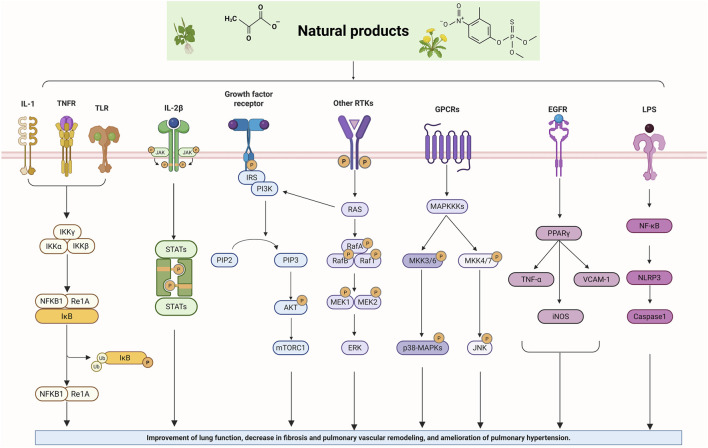
Main signaling pathways and fundamental factors in PH. The major signaling and crosstalk of NF-κB, MAPK, PI3K/AKT, PPARγ, JAK/STAT, and NLRP3 pathways are illustrated. (Created with BioRender-SQ295TYPT7).

Their pharmacological effects are mainly attributed to the following mechanisms by regulating the upstream signaling networks of NLRP3 inflammasome, MAPK, NF-κB, AKT/PI3K, PPARγ, and JAK/STAT, which further reduce the inflammatory cell exudation around the pulmonary arteries, inhibit the expression of pro-inflammatory cytokines, rescue the endothelial dysfunction, and suppress the excessive smooth muscle cell proliferation. The primary signaling pathways and core regulatory factors of pulmonary arterial hypertension are shown in Figure 5. Signaling pathways, including the NLRP3 inflammasome, MAPK, NF-κB, PI3K/Akt, JAK/STAT, PPARγ, Nrf2, and RhoA, play pivotal roles in cellular immune responses, inflammatory processes, metabolic regulation, and cell survival. These pathways can be regulated individually by natural products, yet they also interact extensively with one another. The NLRP3 inflammasome initiates inflammatory responses by recognising intracellular damage-associated molecular patterns (DAMPs) and pathogen-associated molecular patterns (PAMPs), thereby activating downstream MAPK and NF-κB signaling pathways and further promoting cytokine release. NF-κB-regulated gene products, such as IL-6 and IL-8, can activate STAT3, a key transcription factor within the JAK/STAT pathway. Concurrently, NF-κB-regulated gene products enhance NLRP3 inflammasome expression, thereby creating a positive feedback loop. MAPK activation also influences STAT protein phosphorylation and nuclear translocation, thereby modulating JAK/STAT pathway activity and consequently affecting the intensity and duration of inflammatory responses. PPARγ and Nrf2, as pivotal anti-inflammatory and antioxidant regulators, play crucial roles in regulating immune responses and suppressing inflammation. Timely activation of the Nrf2 or PPARγ signaling pathways can inhibit ROS production and suppress NF-κB/MAPK/NLRP3 activation, thereby limiting the inflammatory response. The interplay of these pathways is crucial for elucidating the mechanisms by which natural products modulate inflammatory processes in pulmonary hypertension (PH). Moreover, different bioactive metabolites within the same natural product may exhibit distinct pharmacological effects, thereby highlighting the advantages of natural products in treating PH through multi-component, multi-pathway, and multi-target approaches. This provides a basis for the development of novel therapeutic strategies and the discovery of new drugs.

In summary, the application of natural products in PH therapy provides a broad and promising prospect for new pharmacological research. However, the current study on using NPs to treat PH is still in its early stages and faces many challenges in terms of clinical application. The clinical application of NPs still encounters many problems.

First, research on natural products remains largely confined to cellular and animal studies. Although these *in vivo* experiments provide partial mechanistic insights, the establishment of evidence-based targets and the advancement of natural product research from discovery to clinical application still require the acquisition of reliable patient data (Lythgoe et al., 2016). Current clinical research in this field is characterised by small sample sizes and a lack of large-scale, multicentre, rigorously designed, high-quality trials. Examples include clinical studies investigating sodium tanshinone IIA sulfonate (STS) treatment in PH patients, intravenous apelin therapy in PAH patients (Brash et al., 2018), and the therapeutic effects of nitrate-rich beetroot juice consumption in PAH patients (Henrohn et al., 2018). These studies all featured small sample sizes, and the generalisability and reliability of their findings remain to be fully established. To establish evidence-based targets and advance natural product research from discovery to clinical application, large-scale, multicentre, rigorously designed clinical trials remain essential. For instance, future multicentre randomised controlled trials (RCTs) could provide substantially more robust evidence. Concurrently, evaluating the safety, efficacy, and potential synergistic effects of natural products in combination with existing medications represents a crucial future direction for the development of natural product-based therapies in PH.

Secondly, certain metabolites still present challenges, such as poor oral absorption, low bioavailability, and unclear metabolic pathways. Consequently, it is imperative to explore optimised formulations with enhanced bioavailability through meticulously designed clinical trials. Further efforts may involve the separation and purification of active metabolites to develop biologics with superior pharmacological efficacy. In this regard, the application of nanocarrier drug delivery systems holds promise as an effective approach to elevate the bioavailability of natural metabolites.

Thirdly, pharmacokinetic analysis and safety assessment of natural products remain limited, necessitating further animal/clinical trials to validate their efficacy. Notably, most of the aforementioned studies employed a single-dose regimen. This limitation hinders characterization of the dose–response relationship and precludes estimation of the maximum tolerated dose and the optimal therapeutic dose. Future studies should incorporate dose-ranging (dose-gradient) designs to delineate the effective dose range and therapeutic window, thereby providing more precise dosing guidance for clinical translation. Comprehensive clinical trials are urgently required to investigate the side effects/toxicity and therapeutic outcomes of metabolites. Such research will establish the experimental foundation for optimising formulations, dosing regimens, and combination therapies to achieve therapeutic benefits from natural products.

Fourthly, the model of pulmonary arterial hypertension induced by injecting lily alkaloid (MCT) stands as one of the most widely employed experimental models in current pulmonary hypertension research. The MCT-induced rat model effectively mimics the characteristic pathological alterations of pulmonary arterial hypertension, including vascular remodeling, smooth muscle cell proliferation, endothelial dysfunction, and right ventricular hypertrophy. Nevertheless, despite the MCT model’s extensive application and established research methodology, it possesses certain limitations. Firstly, the MCT model primarily focuses on pathologic alterations induced chemically. While it can mimic some fundamental characteristics of pulmonary arterial hypertension, its pathophysiological processes fall short of replicating the complexity observed in human PAH. Secondly, the current reliance on the MCT model in most animal studies of PAH results in a narrow focus, with insufficient evaluation of other PAH subtypes. Different types of pulmonary hypertension exhibit distinct aetiologies and mechanisms in clinical settings, meaning a single animal model may fail to comprehensively reflect all biological alterations observed in human pathological states. Whilst the MCT model provides a crucial experimental foundation for studying natural products, its limitations in clinical translation are increasingly apparent. Given that most current research remains confined to animal models, with insufficient clinical validation and diverse clinical model support, future studies should expand to encompass a broader range of PAH animal models. Particular emphasis should be placed on models that more accurately mimic human pathological features. By integrating preclinical validation with precision medicine approaches, we can genuinely advance the clinical translation of natural products for PAH treatment.

Fifth, our literature review identified two key limitations in current natural product research: many *in vitro* and *in vivo* studies do not include modern drug (positive) control groups, and investigations of combination therapy with established drugs remain scarce. These gaps hinder objective assessment of the comparative efficacy, safety, and development potential of natural products relative to existing clinical therapies. Accordingly, future work should not be limited to demonstrating standalone efficacy of natural products. Instead, studies should incorporate head-to-head comparisons with standard-of-care drugs and systematically evaluate adjunct or combination regimens. Such comparative and combination studies are essential for identifying candidates with translational potential and informing rational drug development. In addition, several studies provided only *in vitro* data. Although such experiments carry a risk of false-positive results, they can still offer preliminary insights into the potential pharmacological activities of natural products and can guide early mechanistic exploration and candidate selection. However, to achieve meaningful clinical translation, these findings will require rigorous *in vivo* validation followed by clinical evaluation in future studies.

## 7 Outlook and conclusion

In summary, PH is a cardiopulmonary disorder with a bad prognosis and a lack of curative measures that often ends with right ventricular failure. Currently, pharmacotherapies can ameliorate clinical symptoms of PH, but they are not able to target the critical pathological processes, such as pulmonary vascular remodeling, *in situ* thrombosis, and right ventricular dysfunction. Thus, there is still a great challenge in discovering new and effective therapeutic strategies for PH.

Compared with synthetic drugs, NPs generally possess multi-target properties and exhibit low toxicity, which confer them with unique advantages in treating PH. Among these, resveratrol, baicalin, and baicalein appear particularly promising as candidates for pulmonary arterial hypertension (PAH) given their multi-target mechanisms and broad pharmacological profiles, warranting priority evaluation in future research. In recent years, it has been reported that NPs could protect against PH by modulating inflammatory signaling pathways, including NLRP3, MAPK, NF-κB, AKT/PI3K, PPARγ, and JAK/STAT, further reducing perivascular inflammatory infiltration, pro-inflammatory cytokines release, and endothelial dysfunction, as well as smooth muscle cell proliferation. All of these effects interrupt the “inflammation–vascular remodeling” positive feedback loop during PH development, offering new ideas and potential targets for PH treatment.

Looking ahead, natural products hold substantial promise as innovative therapeutic candidates for the treatment of pulmonary hypertension (PH). However, successful translation from the laboratory to clinical application will require both capitalising on their unique advantages and carefully navigating a series of challenges. On the one hand, critical pharmacokinetic issues—including generally low and variable oral bioavailability, complex metabolic pathways, and often unclear dose–exposure–response relationships—need to be addressed through systematic pharmacokinetic/pharmacodynamic studies, rational dose optimisation, and the development of optimised delivery systems (e.g., nano- or microparticle and inhalation formulations) to enhance targeted pulmonary exposure while minimising systemic toxicity. In parallel, rigorously designed long-term preclinical and clinical studies are required to characterise the safety profiles of natural products, with particular emphasis on batch-to-batch consistency, potential botanical drug–drug interactions, and organ-specific adverse reactions under chronic administration. Building upon this foundation, multicentre, large-sample, long-term follow-up clinical trials will be needed to systematically evaluate their efficacy and safety profiles. Furthermore, future research should not only examine the efficacy of individual metabolites but also explore rational combination strategies, using natural products as adjuncts to existing standard therapies (such as endothelin receptor antagonists, phosphodiesterase-5 inhibitors, and prostacyclin analogues) to achieve additive or synergistic benefits. This approach may allow dose reduction of conventional drugs and mitigate adverse effects. Overall, efforts should focus on systematically addressing critical issues such as bioavailability, optimal dosing, long-term safety, and quality control. This will help advance their clinical translation and standardised application in the treatment of pulmonary arterial hypertension.

## Glossary

PH: Pulmonary hypertension
PAH: Pulmonary arterial hypertension
PVR: Pulmonary vascular remodeling
sGC: Soluble guanylate cyclase
PDE5: Phosphodiesterase type 5
ERAs: Endothelin receptor antagonists
NPs: Natural products
PASMCs: Pulmonary arterial smooth muscle cells
EndMT: Endothelial-to-mesenchymal transition
NO: Nitric oxide
eNOS: Endothelial nitric oxide synthase
ET-1: Endothelin-1
cGMP: Cyclic guanosine monophosphate
GTP: Guanosine triphosphate
PGI2: Prostacyclin
ROS: Reactive oxygen species
ECM: Extracellular matrix
NF-κB: Nuclear factor-κB
TNF-α: Tumor necrosis factor-alpha
Gyp: Gypenoside
Ost: Osthole
BAI: Baicalein
BE: Baicalin
18β-GA: 18β-Glycyrrhetinic acid;
HPASMC: Human Pulmonary Artery Smooth Muscle Cells
GSP: Grape seed proanthocyanidins
TRAF2: Tumor necrosis factor receptor-associated factor 2
SphK1: Sphingomyelin kinase 1
S1P: Sphingomyelin 1-phosphate
Gen: Genistein
HIF-1α: Hypoxia-inducible factor-1α
SDF-1: Stromal cell-derived factor-1
CXCR4: Chemokine receptor 4
STAT3: Signal Transducer and Activator of Transcription 3
GSK3β: Glycogen synthase kinase 3β
GSP: Grape seed proanthocyanidins
TLRs: Toll-like receptors
TGF-β1: Transforming growth factor beta-1
MCP-1: Monocyte chemotactic protein-1
PTE: Pulmonary thromboembolism
ASIV: Astragaloside IV
MMP9: Matrix metalloproteinase-9
